# Heterogeneity of intrinsic plasticity in cerebellar Purkinje cells linked with cortical molecular zones

**DOI:** 10.1016/j.isci.2021.103705

**Published:** 2021-12-28

**Authors:** Nguyen-Minh Viet, Tianzhuo Wang, Khoa Tran-Anh, Izumi Sugihara

**Affiliations:** 1 Department of Systems Neurophysiology, Graduate School of Medical and Dental Sciences, Tokyo Medical and Dental University, Tokyo, Japan; 2 Center for Brain Integration Research, Tokyo Medical and Dental University, Tokyo, Japan

**Keywords:** Neuroscience, Cellular neuroscience

## Abstract

In the cerebellar cortex, heterogeneous populations of Purkinje cells (PCs), classified into zebrin (aldolase C)-positive (Z+) and -negative (Z-) types, are arranged into separate longitudinal zones. They have different topographic neuronal connections and show different patterns of activity in behavior tasks. However, whether the zebrin type of PCs directly links with the physiological properties of the PC has not been well clarified. Therefore, we applied *in vitro* whole-cell patch-clamp recording in Z+ and Z- PCs in vermal and hemispheric neighboring zebrin zones in zebrin-visualized mice. Intrinsic excitability is significantly higher in Z- PCs than in Z+ PCs. Furthermore, intrinsic plasticity and synaptic long-term potentiation are enhanced more in Z- PCs than in Z+ PCs. The difference was mediated by different modulation of SK channel activities between Z+ and Z- PCs. The results indicate that cellular physiology differentially tunes to the functional compartmentalization of heterogeneous PCs.

## Highlights

• Zebrin-negative Purkinje cells are more excitable than zebrin-positive ones
• Zebrin-negative Purkinje cells also have higher plasticity of intrinsic excitability
• The difference is mediated by different modulation of SK channel activities
• Purkinje cell physiology differentially tunes to the cerebellar compartmentalization

## Graphical abstract

**Figure:**
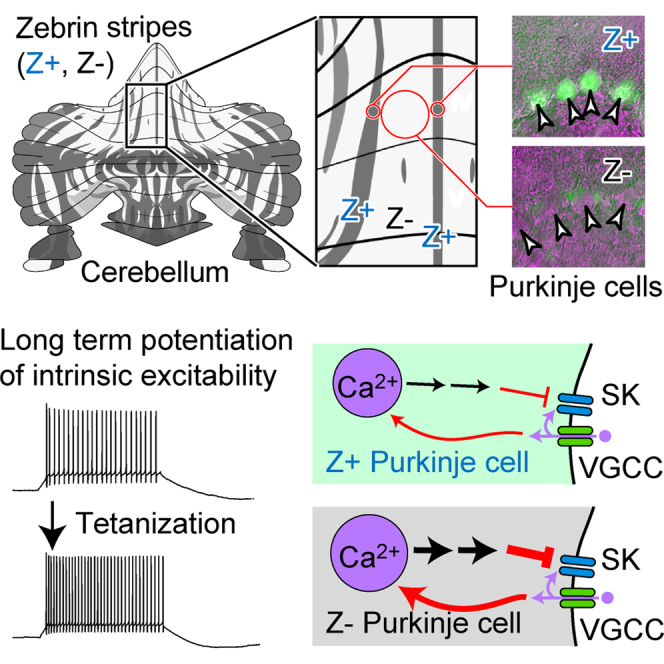

## Introduction

Mechanisms of long-term information storage in the neuronal network include plasticity of a neuron's intrinsic excitability—intrinsic plasticity (Abraham, 2008; Titley et al., 2017), i.e., change in the ability of repetitive firing in response to excitatory inputs. Neuronal intrinsic plasticity plays an essential role in the hippocampus for fear conditioning (Titley et al., 2017; McKay et al., 2009) and trace eyeblink conditioning (McEchron and Disterhoft, 1997; Disterhoft and Oh, 2006) in nucleus accumbens for cocaine addiction (Kourrich et al., 2015), and in the deep cerebellar nuclei for delay eyeblink conditioning (Canto et al., 2018; Wang et al., 2018). In membrane potential recording, neuronal intrinsic plasticity is often associated with changes in the intensity of afterhyperpolarization (AHP), which follows an action potential or other types of depolarization with various (fast, medium, and slow) time courses (Disterhoft and Oh, 2006) and affects the following depolarization and action potential generation. Voltage-activated or calcium-activated potassium channels underlie the fast AHP and some of the medium AHP (Disterhoft and Oh, 2006).

PCs are the sole output neuron in the cerebellar cortex. Thus, their excitability and plasticity are deeply involved in cerebellar functions, including control and adaptation of motor performance (Ito, 2012). Although PCs fire spontaneously, their firing frequency and pattern are dependent on and modulated by their excitability and various synaptic inputs (Zhou et al., 2014; Xiao et al., 2014; De Zeeuw and Ten Brinke, 2015). Their inhibitory output then controls the firing pattern of target nuclear neurons (Person and Raman, 2011). Thus, the robust intrinsic plasticity of PCs, as reported in *in vivo* and *in vitro* preparation (Schreurs et al., 1998; Belmeguenai et al., 2010; Grasselli et al., 2016; Shim et al., 2018), can significantly affect the PC output and the activity of cerebellar nuclear neurons (Canto et al., 2018; Person and Raman, 2011; Wang et al., 2018).

Noticeably, PCs are composed of heterogeneous populations that express many molecules at different levels (Cerminara et al., 2015; Hawkes, 2014). Since zebrin (zebrin II, aldolase C) is the representative of such molecules, PCs are generally classified into zebrin-positive (Z+) and zebrin-negative (Z-) populations (Brochu et al., 1990; Pijpers et al., 2006; Sugihara and Shinoda, 2004). Z+ and Z- PCs are distributed in alternate longitudinal zones to project to a particular subarea of the cerebellar nuclei and be innervated by a particular subarea of the inferior olive according to the precise topographical connection pattern (Cerminara et al., 2015; De Zeeuw and Ten Brinke, 2015; Pijpers et al., 2006; Sugihara and Shinoda, 2004). Thus, heterogeneous PCs located in different longitudinal zones belong to distinct functional networks (Aoki et al., 2019; Sugihara and Shinoda, 2004; Suzuki et al., 2012; Tran-Anh et al., 2020). Indeed, Purkinje cells (PCs) in different longitudinal zones show distinct activity patterns in the anesthetized state (Sugihara et al., 2007; Tsutsumi et al., 2015) and during behaving tasks with rewards (Kostadinov et al., 2019; Tsutsumi et al., 2019).

Many other molecules are also expressed heterogeneously in PCs in the manner linked with the zebrin expression (Cerminara et al., 2015; Hawkes, 2014), suggesting the possibility that various physiological properties are affected differently in heterogeneous populations of PCs. In synaptic transmissions in and around PCs, weaker glutamatergic transmission in extrasynaptic areas due to higher expression of the glutamate transporter EAAT4 in Z+ PCs than in Z- PCs has been reported (Malhotra et al., 2021; Wadiche and Jahr 2005). Concerning excitability of PCs, Z- PCs show higher firing frequencies than in Z + PCs in *in vivo* and *in vitro* preparations (Nguyen-Minh et al., 2019; Wu et al., 2019; Xiao et al., 2014; Zhou et al., 2014). The higher excitability in Z- PCs is partly mediated by transient receptor potential cation channel C3 (TRPC3), which is expressed less highly in Z+ PCs than in Z- PCs in the vermis (Wu et al., 2019). However, these comparative studies of excitability randomly sampled PCs in various lobules and zones (Xiao et al., 2014; Zhou et al., 2014) or compared PCs between lobule III and lobule X, which are mainly occupied by Z- zones and Z+ zones, respectively (Wu et al., 2019). So far, few studies (Malhotra et al., 2021; Nguyen-Minh et al., 2019) have compared PCs in neighboring Z+ and Z- zones in the same lobule to eliminate possible effects of lobular differences. Furthermore, the intrinsic plasticity of PCs has not been compared between Z+ and Z- PCs.

Therefore, we aimed at examining intrinsic excitability, plasticity, and related parallel fiber (PF)-PC synaptic properties from Z+ and Z- PCs identified in neighboring zebrin zones in vermal lobule IV-V and hemispheric crus II. For this purpose, we used slice preparation from young AldocV and Rgs8-EGFP mice (P16–P24), which allows identification of the zebrin type in PCs and eliminates external topographic inputs to PCs.

## Results

### Z+ and Z- PCs in the sagittal slice preparation

**Figure 1:**
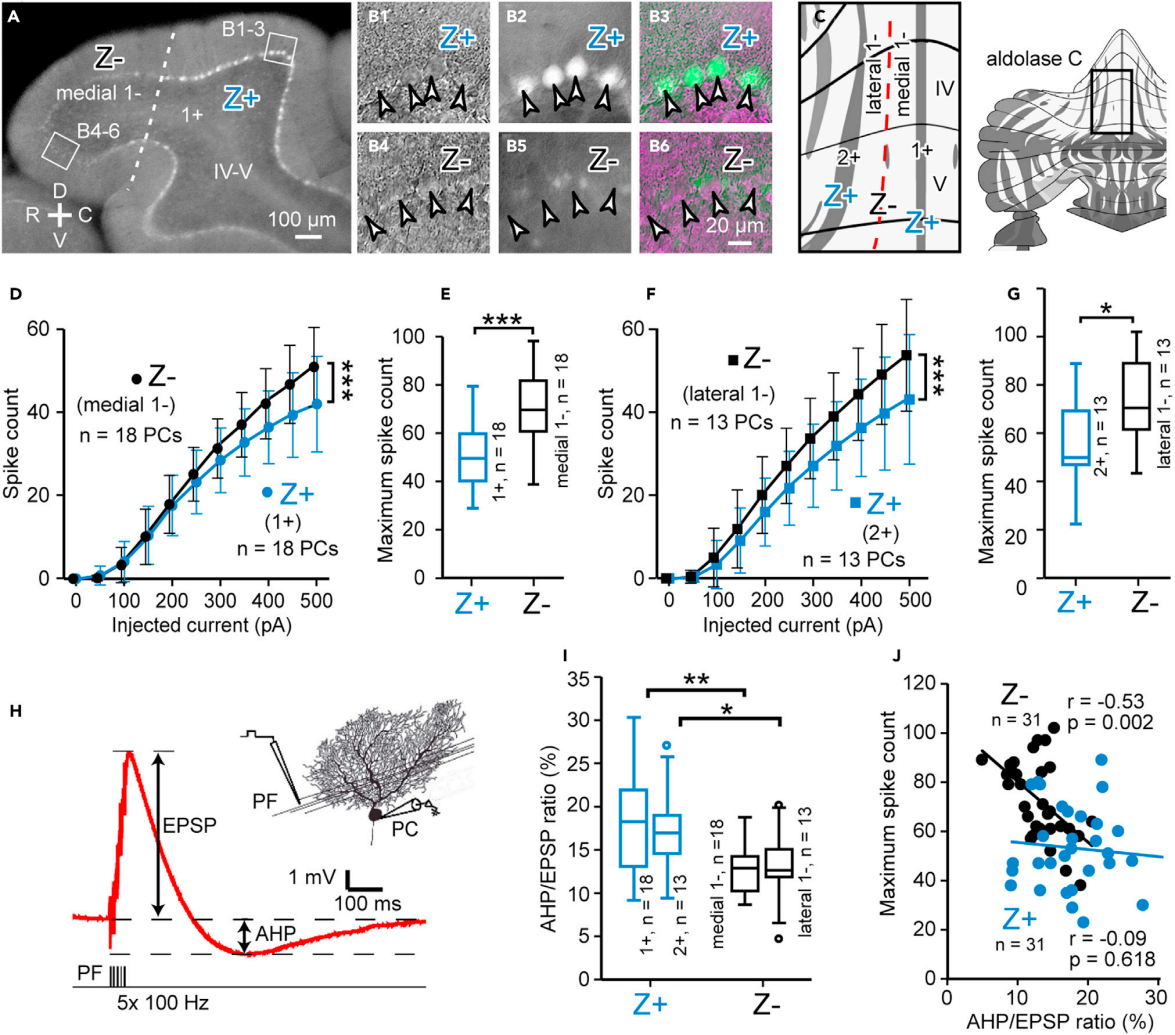
Comparison of the intrinsic excitability and afterhyperpolarization (AHP) between Z+ and Z- PCs in neighboring zones in vermal lobule IV-V (A) Identification of Z+ and Z- zones in the sagittal slice preparation in the nearly midsagittal plane of vermal lobules IV-V in the AldocV mouse at postnatal day 20 (P20). The dashed lines indicate the boundary of zones 1+ and 1-. Squares indicate the areas for (B). (B) Z+ and Z- PC under IR-DIC (B1, 4), epifluorescence (B2, B5), and merged image (B3, B6) in the slice preparation. (C) Recording area of this study, located (square in the right inset) within the scheme of zebrin zones in the unfolded cerebellar cortex (Sarpong et al., 2018). (D and F) Plot of spike-current relationships, i.e., the spike count measured during 500-ms square current injection of various intensities under current-clamp mode in Z+ and Z- PCs in neighboring zones. (E and G) Comparison of the maximum spike count between Z+ and Z- PCs in neighboring zones. (H) Illustration of measurement of the subthreshold AHP following EPSP of the parallel fiber-Purkinje cell synapse. (I) Comparison of the AHP intensity (ratio of the AHP amplitude to the EPSP amplitude) between Z+ and Z- PCs in neighboring zones. (J) The relationship between the AHP intensity and maximum spike count in Z+ and Z- PCs. Regression analysis was performed to get Person's r values and p values. Data were obtained in 18 Z+ PCs in zebrin zone 1+, 18 Z- PCs in lateral zone 1-, 13 Z- PCs in lateral zone 1-, and 13 Z+ PCs zone 2+ in lobule IV-V in (D–J). Data are represented as mean ± standard deviation in (D and F) and Tukey method box and whisker graphs in (E, G, and I). We used two-way ANOVA with repeated measures (D, F(1,395) = 13.88, p = 0.00022, n = 18, 18; F, F(1,285) = 28.2, p = 0.00000, n = 13, 13) and unpaired Student's t test (E, t(34) = -3.91, p = 0.0004, n = 18; G, t(24) = -2.64, p = 0.014, n = 13; I, 1+/medial 1-, t(34) = 3.39, p = 0.002, n = 18; 2+/lateral 1-, t(24) = 2.48, p = 0.021, n = 13) to test for the significant difference. ∗∗∗p < 0.001, ∗∗p < 0.01, ∗p < 0.05. Pearson's r test was used in (J). Abbreviations: IV-V, lobule IV-V; 1+, 1-, 2+, zebrin zones 1+, 1- and 2+; C, caudal; D, dorsal; R, rostral; V, ventral.

We performed whole-cell current-clamp and voltage-clamp recordings in Z+ and Z- PCs in two pairs of neighboring zones (zone 1+ and medial zone 1-, and lateral zone 1- and zone 2+) in vermal lobule IV-V (Figures 1A–1C) in the sagittal slice prepared from heterozygous AldocV mice (Fujita et al., 2014) at postnatal day (P) 16–24. The unique zebrin zone pattern with narrow midline positive zone 1+, wide negative zone 1-, and narrow positive zone 2+, which is located at about 400 μm to the midline, slightly wider than 1+ and slightly tilted from the parasagittal plane, facilitated the identification of these zones in the sagittal slice preparation (Figure 1C).

### Intrinsic excitability is stronger in Z- PCs than in Z+ PCs

To examine the intrinsic excitability of PCs, we plotted the number of spikes evoked by 500-ms current injection against the intensity of the injected current for Z+ and Z- PCs under current-clamp mode. The increase of the spike count was similar between Z+ and Z- PCs for small current injections (0–200 pA), whereas it became higher in Z- PCs than in Z+ PCs for higher current injections (300–500 pA), in both pairs of neighboring zones (1+ and medial 1-, F(1,395) = 13.88, p = 0.00022, n = 18, 18, medial 1- and 2+, F(1,285) = 28.2, p = 0.00000, n = 13, 13, two-way ANOVA with repeated measurement, Figures 1D and 1F). With even larger current injections (500–1,000 pA), the spike count generally showed further increase, saturation, and decrease due to intrinsic depolarization block (Figures S1A and S1C). The maximum spike count per 500 ms in the range of 0–1,000 pA current injection was significantly higher in Z- PCs than in Z+ PCs in zones 1+ and medial 1- (mean, 70.9 versus 51.6, t(34) = -3.91, p = 0.0004, unpaired t test, Figure 1E), as well as in zones 2+ and lateral 1- (mean 75.0 versus 55.2, t(24) = -2.64, p = 0.014, Figure 1G). The duration of spike firing during current injection, “firing duration,” and the current injection intensity that elicits the largest number of spikes, “maximum firing current,” also reflect the intrinsic depolarization block. The firing duration and maximum firing current were significantly shorter and smaller, respectively, in Z+ PCs than in Z- PCs in both areas (Figures S1B and S1D). In the present study, we did not observe significant differences between Z+ and Z- PCs in the input resistance (80.28 ± 13.97 and 76.89 ± 10.55 MΩ, average ± standard deviation, in 31 Z+ and 31 Z- PCs, respectively), or rheobase current (112.90 ± 31.53 and 119.35 ± 33.36 pA), as observed in our previous study in lobule VIII (Nguyen-Minh et al., 2019).

These results indicated that Z- PCs have higher excitability than Z + PCs in neighboring zebrin zones in vermal lobule IV-V. It was in line with similar findings obtained in lobule VIII (Nguyen-Minh et al., 2019) and findings of higher simple spike firing rate in Z- PCs than in Z+ PCs in various areas of the rat and mice *in vivo* cerebellum (Wu et al., 2019; Xiao et al., 2014; Zhou et al., 2014).

### Subthreshold AHP was more correlated with excitability in Z- PCs than in Z+ PCs

The different intrinsic excitability between Z+ and Z- PCs indicates some difference in depolarization-evoked ionic conductance between these PCs. The AHP evoked by a spike or burst of spikes and the AHP evoked by subthreshold depolarization (“subthreshold AHP”) reflect such conductance. Furthermore, subthreshold AHP can be measured more simply and accurately than the other (Belmeguenai et al., 2010; Grasselli et al., 2016; Paukert et al., 2010; Schreurs et al., 1998). We, therefore, compared subthreshold AHP to elucidate mechanisms for different repetitive spike firing responses between Z+ and Z- PCs.

Subthreshold AHP was observed under current-clamp mode following the EPSP depolarization of the PF-PC synapse evoked by stimulating PFs with a train of five stimuli at 100 Hz (Figure 1H). We then measured the AHP/EPSP ratio, rather than the AHP amplitude itself, to extract the size of the subthreshold AHP by reducing noise. The AHP/EPSP ratio was significantly higher in Z+ PCs than in Z- PCs in zones 1+ and medial 1- (mean,18.2% versus 12.9%, t(34) = 3.29, p = 0.002, unpaired t test, n = 18,18, Figure 1I), as well as in zones 2+ and lateral 1- (mean, 17.2% versus 13.0%, t(24) = 2.48, p = 0.02, unpaired t test, n = 13,13, Figure 1I). The two areas had the same range of data distribution; combining these data gave even a more significant difference in the AHP/EPSP ratio between Z+ and Z- PCs (t(60) = 4.25, p = 0.00008, unpaired Student's t test, n = 31,31). The maximum spike count in the range of 0–1,000 pA current injection was plotted against the AHP/EPSP ratio for Z+ and Z- PCs to determine the correlation between the subthreshold AHP and the intrinsic excitability of individual PCs (Figure 1J). The AHP/EPSP ratio was correlated negatively with the maximum spike count in Z- PCs (Pearson's r = −0.53, p = 0.002, Figure 1J). On the contrary, the AHP/EPSP ratio was not significantly correlated with the maximum spike count in Z+ PCs (Pearson's r = −0.09, p = 0.62, Figure 1J).

The results as a whole indicated that the underlying ionic conductance mechanisms that determined the firing frequency was partly different between Z+ and Z- PCs. Although the AHP/EPSP ratio was about 1.36 times larger in Z+ PCs than in Z- PCs, the AHP/EPSP ratio seemed to reflect the ionic conductance that contributes to the firing frequency in Z- than in Z + PCs.

The results showed that data obtained from Z+ PCs in zone 1+ and zone 2+ had similar values and that data obtained from Z- PCs in medial zone 1- and lateral zone 1- also had similar values. This tendency ran through all the remaining experiments in the present study. Therefore, we combine Z+ PCs from zones 1+ and 2+ and Z- PCs from medial and lateral zone 1- of lobule IV-V in statistical analyses in succeeding sections.

**Figure 2:**
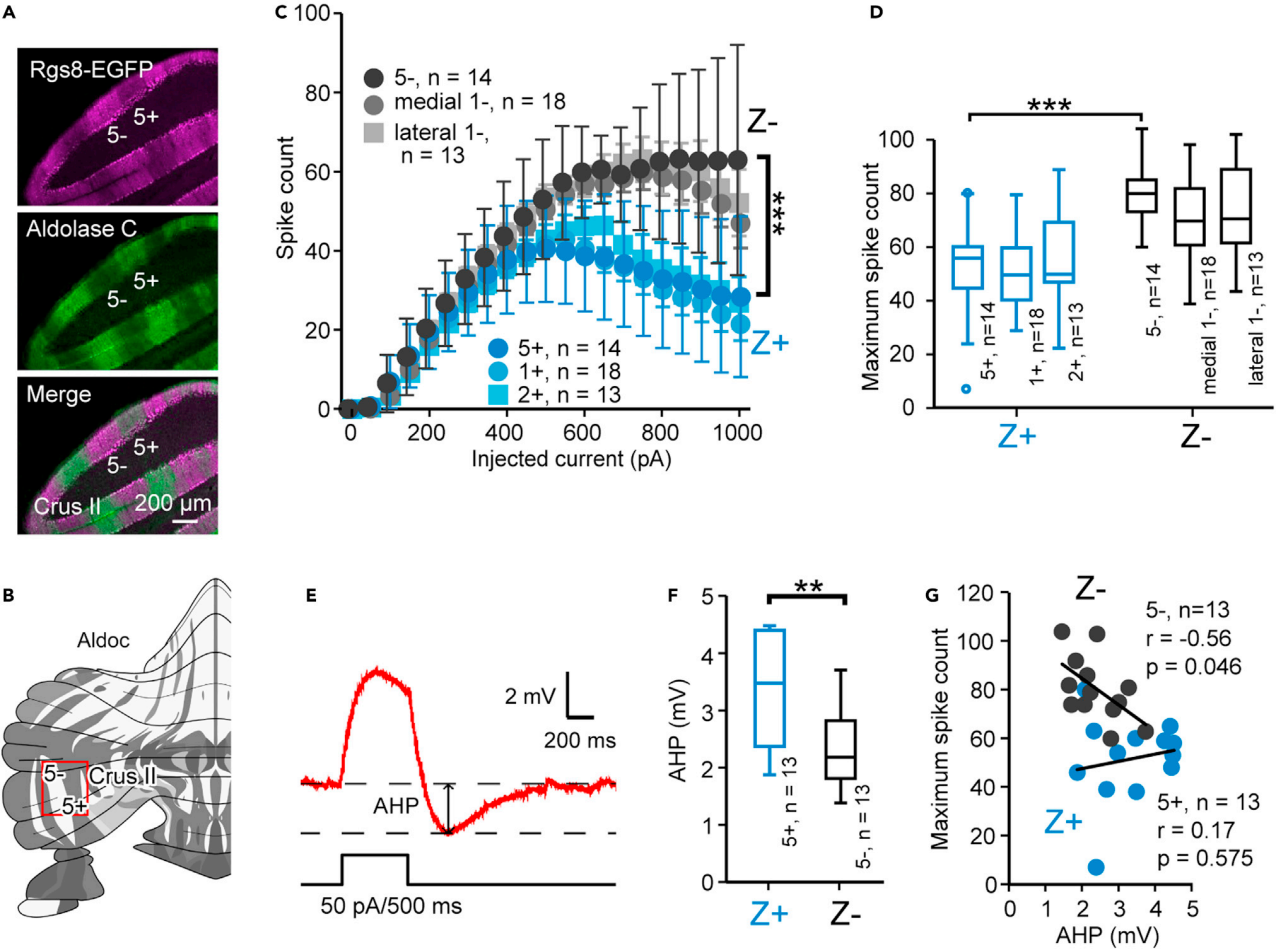
Preservation of the different properties between Z+ and Z- PCs in Rgs8EGFP mice (A) Immunohistological evaluation of the striped pattern of EGFP expression. EGFP expression (left top), Aldolase C immunostaining (left center), and merged image (left bottom) of a coronal section of the caudal cerebellum, showing expression of EGFP in Z- (= aldolase C-negative) PCs. (B) Recording area (zones 5+ and 5- in crus II) indicated by the red square in the unfolded scheme of the cerebellum. This area was selected since zone 1+ and 2+, narrow EGFP-negative stripes in these mice, were difficult to locate in lobules IV-V. (C) Spike-current relationships measured with 500-ms square current injection of various intensities under current-clamp mode in 14 Z+ and 14 Z- PCs in zones 5+ and 5- in crus II, compared with those in zones 1+, medial 1-, lateral 1-, 2+ in lobule IV-V. (D) Comparison of the maximum spikes count between Z+ and Z- PCs, obtained in the measurement of (C). (E) Current injection protocol to measure the intensity of the AHP evoked by a subthreshold depolarization. (F) Comparison of subthreshold AHP amplitude between Z+ and Z- PCs. AHP of Z- PCs (2.16 ± 0.19 mV) was significantly smaller than that of Z + PCs (3.32 ± 0.27 mV) (∗∗p < 0.01, n = 13 in each group). (G) Relationship between the maximum spike count (as in D) and the subthreshold AHP amplitude (as in F) for Z+ and Z- PCs. No correlation was observed in Z + PCs, whereas a negative correlation was observed in Z- PCs. Data are represented as mean ± standard deviation in (C) and Tukey method box and whisker graphs in (D, F). The significant difference was tested with two-way ANOVA with repeated measures in (C, 5+/5-, F(1,546) = 148.51, p = 0.0000, n = 14, 14) and unpaired Student t test in (D, 5+/5-, t(26) = -5.074, p = 0.0000, n = 14, 14; F, t(24) = 2.86, p = 0.0087, n = 13, 13). ∗∗∗p < 0.001, ∗∗p < 0.01. Pearson's r test was used in (G).

By considering the possibility that the genetic manipulation in Aldoc-Venus mice may affect physiological properties of PCs, we also used different transgenic mice with cerebellar zone visualization (Rgs8-EGFP) to confirm different properties between Z+ and Z- PCs. In Rgs8-EGFP mice, in which Z- PCs express fluorescence, the narrow fluorescence-negative zones 1+ and 2+ were challenging to locate in lobule IV-V. Therefore, we used zones 5+ and 5- in crus II in slice preparations cut from the lateral cerebellum of Rgs8-EGFP mice (Figures 2A and 2B). The spike-current relationship (Figure 2C), maximum spike count (Figure 2D), subthreshold AHP intensity (Figures 2E and 2F), and the correlation between the subthreshold AHP intensity and maximum spike count (Figure 2G) showed significantly different values between Z+ and Z- PCs, nearly to the same extent as observed in lobule IV-V in AldocV mice.

### LTP of intrinsic excitability is more enhanced in Z- zones than in Z+ zones

**Figure 3:**
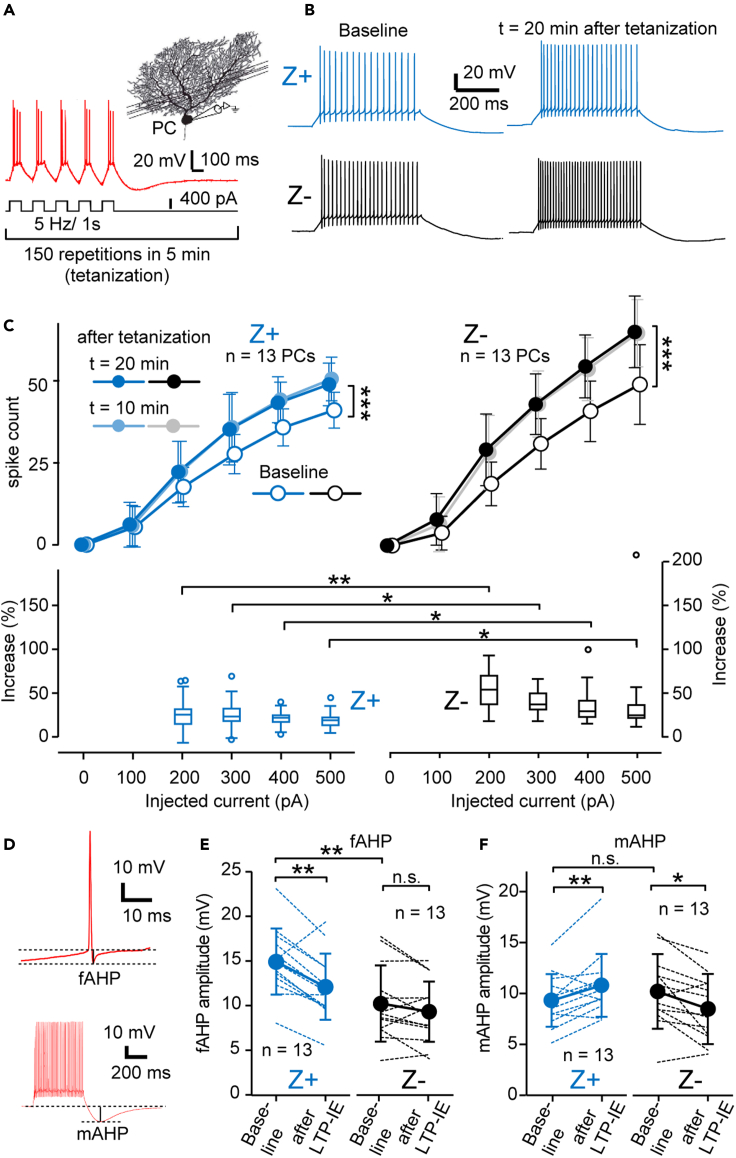
Comparison of the intrinsic plasticity between Z+ and Z- PCs in neighboring zones (A) Illustration of the direct PC stimulation (tetanization) protocol induces the long-term potentiation of the intrinsic excitability (LTP-IE) or intrinsic plasticity. (B) Spike response to square current injection (300 pA and 500 ms) before and 20 min after giving the LTP-IE protocol in Z+ and Z- PCs. (C) Change the spike-current relationship in Z+ (top left) and Z- (top right) PCs. The number of spikes during 500-ms square current injection of various intensities (100–500 pA) was plotted against the intensity of the injected current, measured before (baseline) and 10 and 20 min after the LTP-IE protocol Then, we calculated the spike count increase (bottom) from the measurements before and 20 min after the LTP-IE protocol. (D) Illustration of measurement of fAHP and mAHP. (E and F) Change of the fAHP (E) and mAHP (F) in LTP-IE. Amplitudes of the fAHP (E) and mAHP (F) in individual PCs (dashed lines) and in the average (solid line) were compared before and 20 min after the LTP-IE protocol in 13 Z+ (left graph in each panel) and 13 Z- (right graph in each panel) PCs. Data are represented as mean ± standard deviation in (C top, E and F) and Tukey method box and whisker graphs in (C bottom). The significant difference was tested with two-way ANOVA with repeated measures (C top, before and 20 min after; Z+, F(1,144) = 20.46, p = 0.00001, n = 13, 13; Z-, F(1,144) = 49.54 p = 0.00000, n = 13, 13), Mann-Whitney U test (C bottom; n1 = 13, n2 = 13; 200 pA, W = 21, p = 0.001; 300 pA, W = 35, p = 0.012; 400 pA, W = 39, p = 0.021; 500 pA, W = 40.5, p = 0.026), unpaired Student's t test (E, Z+/Z-baseline, t(24) = 3.38, p = 0.0024, n = 13, 13; F, Z+/Z-baseline, t(24) = -0.760, p = 0.455, n = 13, 13), and paired Student's t test (E, Z+, t(12) = 4.11, p = 0.0014, n = 13; Z-, t(12) = 1.28, p = 0.224, n = 13; F, Z+, t(12) = -2.8, p = 0.015, n = 13; Z-, t(12) = 3.18, p = 0.008, n = 13). ∗∗∗p < 0.001, ∗∗p < 0.01, ∗p < 0.05. All recorded PCs were located in zones 1+, 1-, and 2+ in lobule IV-V.

The intrinsic excitability is potentiated permanently by tetanizing stimulation (long-term potentiation of intrinsic excitability or LTP-IE) in PCs (Belmeguenai et al., 2010; Grasselli et al., 2016; Shim et al., 2018). However, the comparison of this property has not been made between Z+ and Z- PCs. In the present study, we compared the change in the intrinsic excitability produced by tetanizing stimulation between Z+ and Z- PCs in zones 1+, 1-, and 2+ in vermal lobule IV-V in AldocV mice under current-clamp. After giving the tetanizing stimulation, a square current injection of 100–1,000 pA evoked a significantly larger number of spikes than before (Figures 3A and S2), representing LTP-IE in both Z+ and Z- PCs (Figure 3B). Meanwhile, LTP-IE, once evoked by tetanization, was stable for at least 20 min, as observed in the upward shift of the spike-current relationship in both Z+ and Z- PCs (“baseline,” “10 min,” and “20 min” after tetanization, Figure 3C). This enhancement of the spike number 20 min after tetanization was statistically significant in both Z+ (Figure 3C top left, F(1,144) = 20.46, p = 0.00001, two-way ANOVA with repeated measure, n = 13) and Z- PCs (Figure 3C top right, F(1,144) = 49.54 p = 0.00000, n = 13). We also confirmed that the spike-current relationship was stable among “baseline,” “10 min,” and “20 min” in both Z+ and Z- PCs without tetanization (Figure S3).

Next, we compared the enhancement in the spike-current relationship by the tetanization protocol between Z+ and Z- PCs. The enhancement was 1.5- to –3-fold stronger in Z- PCs than in Z+ PCs at 200, 300, 400, and 500 pA current injections (Figure 3C bottom, p = 0.0012, p = 0.012, p = 0.02, p = 0.026, respectively, Mann-Whitney U test, n = 13). Therefore, this indicated a higher degree of LTP-IE in Z- PCs than in Z+ PCs.

According to the spike-current relationship (Figures 1D and 1F), the tetanization with 400 pA current injection may have caused about 1.2 times more spikes in Z- PCs than in Z + PCs. However, the degrees of spike enhancement were 1.5–3 times higher in Z- PCs than in Z + PCs. Therefore, other than spike number differences, some particular mechanisms were likely to underlie the different degrees of LTP-IE between Z+ and Z- PCs.

### Distinct modulation of different AHP phases underlying the LTP-IE in Z+ and Z- PCs

Intrinsic plasticity occurs by the AHP depression in PCs (Belmeguenai et al., 2010; Disterhoft and Oh, 2006; Grasselli et al., 2016). Since different phases of AHP are modulated separately and involved in changing the intrinsic excitability (Disterhoft and Oh, 2006), we next examined the relationship between intrinsic plasticity and changes in different phases of AHP in Z+ and Z- PCs. We classified the AHP into three types, i.e., fast, medium, and slow AHPs (fAHP, mAHP, and sAHP), according to Disterhoft and Oh (2006). These AHP classes were observed during and after spike responses evoked by 500-ms square-shaped current injections under current-clamp mode. The fAHP indicate the hyperpolarization occurring after a single spike with a latency of less than a few milliseconds, whereas the mAHP and sAHP indicate the peak (latency, 100–200 ms) and decaying phase (at a latency of 500 ms), respectively, of the hyperpolarization occurring after a burst of spikes (Figures 2D and S3A). These different phases of AHP reflect different underlying mechanisms (ionic channels and ionic pumps, Disterhoft and Oh, 2006) and contribute to intrinsic plasticity differently (Belmeguenai et al., 2010).

We compared amplitudes of fAHP, mAHP, and sAHP between the baseline period before giving the LTP-IE protocol (tetanizing stimulation) and 20 min after the LTP-IE protocol in Z+ and Z- PCs under current-clamp mode (Figures 3E, 3F, and S4B). Z+ PCs showed a decrease in fAHP (Figure 3E, left, t(12) = 4.11, p = 0.0014, paired Student's t test, n = 13), an increase in mAHP (Figure 3F, left, t(12) = -2.8, p = 0.015), and no significant change in sAHP (Figure S4B, left, t(12) = -1.175, p = 0.263). However, Z- zones PCs showed a decrease in mAHP (Figure 3F, right, t(12) = 3.18, p = 0.008, paired Student's t test, n = 13) and no significant change in fAHP (Figure 3E, right, t(12) = 1.28, p = 0.224) or in sAHP (Figure S4B, right, t(12) = -0.882, p = 0.395). Our results indicated that the LTP-IE protocol (tetanizing stimulation) modulates AHP differently in Z+ and Z- PCs. The decrease in fAHP and the decrease in mAHP were likely to be responsible for increasing the spike number, i.e., the intrinsic plasticity, in Z+ and Z- PCs, respectively. The subthreshold AHP, which correlated to the maximum spike frequency in Z- PCs in the preceding section, was similar to the mAHP in the time course.

### Downregulation of SK2 channels mediates intrinsic plasticity in Z- PCs

Previous studies reported that SK2 channels, an isoform of small conductance Ca^2+^-activated K channels (SK channels), play the leading role in producing intrinsic plasticity in PCs (Belmeguenai et al., 2010; Grasselli et al., 2016), although these studies have not distinguished zebrin types of PCs. Therefore, to determine the ionic mechanism underlying the plastic change in the intrinsic excitability in Z+ and Z- PCs, we applied apamin (100 nM, SK2 channel blocker, Belmeguenai et al., 2010) and iberiotoxin (50 nM, maxi calcium-activated, BK, channel blocker, Belmeguenai et al., 2010) in the bath solution and replicated LTP-IE experiments.

**Figure 4:**
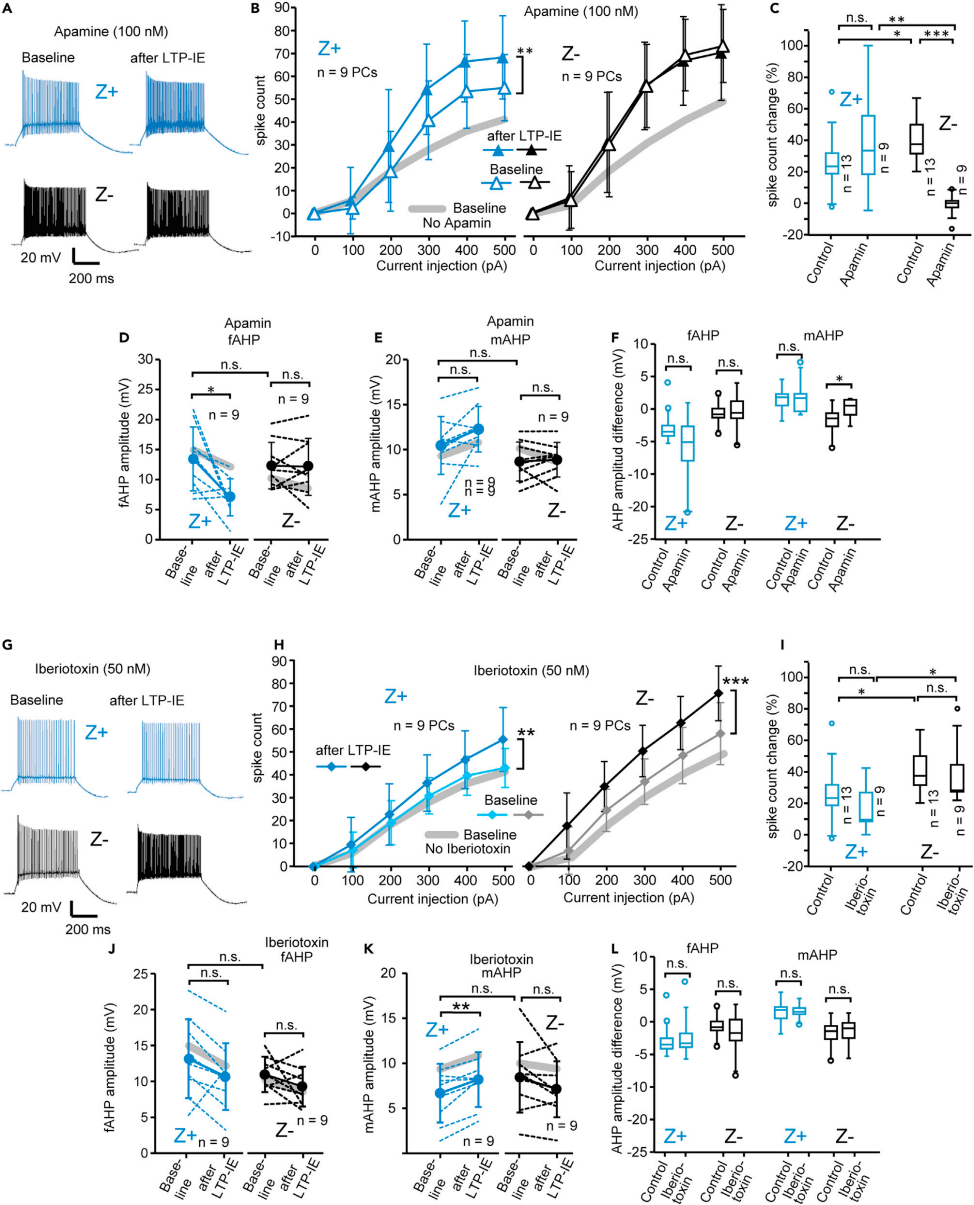
Intrinsic plasticity examined under the administration of blockers of Ca-activated potassium channels in Z+ and Z- PC (A) Spike response to square current injection (300 pA and 500 ms) before and 20 s after giving the LTP-IE protocol in Z+ and Z- PCs under apamin (100 nM) administration. (B) The spike-current relationship before and 20 s after giving the LTP-IE protocol in Z+ and Z- PCs under apamin. The thick gray line is the spike-count relationship before the LTP-IE protocol without apamin (as shown in Figure 3C) for comparison. (C) Spike count change calculated from the measurements with 300-pA current injection before and 20 min after the LTP-IE protocol in the control condition (Figure 3C), and under apamin. (D and E) Change in fAHP (D) and mAHP (E) in the LTP-IE protocol under apamin. Amplitudes of the fAHP (D) and mAHP (E) in individual PCs (dashed lines) and the average (solid line) were compared before and 20 min after the LTP-IE protocol in 9 Z+ (left graph in each panel) and 9 Z- (right graph in each panel) PCs. Pale gray lines indicate the average change under the control condition (as shown in Figures 3E and 3F). (F) AHP amplitude change obtained for each PC by subtracting the AHP amplitude under baseline from the AHP amplitude 20 min after the LTP-IE protocol. (G) Spike response to square current injection (300 pA and 500 ms) before and 20 s after giving the LTP-IE protocol in Z+ and Z- PCs under iberiotoxin (50 nM) administration. (H) The spike-current relationship before and 20 s after giving the LTP-IE protocol in Z+ and Z- PCs under iberiotoxin. The thick gray line is the spike-count relationship before the LTP-IE protocol without iberiotoxin (as shown in Figure 3C) for comparison. (I) Spike count change calculated from the measurements with 300-pA current injection before and 20 min after the LTP-IE protocol in the control condition (Figure 3C), and under iberiotoxin. (J and K) Change in fAHP (J) and mAHP (K) in the LTP-IE protocol under iberiotoxin. Amplitudes of the fAHP (J) and mAHP (K) in individual PCs (dashed lines) and the average (solid line) were compared before and 20 min after the LTP-IE protocol in 9 Z+ (left graph in each panel) and 9 Z- (right graph in each panel) PCs. Pale gray lines indicate the average change under the control condition (Figures 3E and 3F). (L) AHP amplitude change obtained for each PC by subtracting the AHP amplitude under baseline from the AHP amplitude 20 min after the LTP-IE protocol. Data are represented as mean ± standard in (B, D, E, H, J, and K) and Tukey method box and whisker graphs in (C, F, I, and L). The significant difference was tested with two-way ANOVA with repeated measures (B, Z+, F(1,96) = 9, p = 0.003, n = 9, 9; Z-, F(1,96) = 0.04 p = 0.85, n = 9, 9; H, Z+, F(1,96) = 7.18, p = 0.0087, n = 9, 9; Z-, F(1,96) = 29.17, p = 0.0005, n = 9, 9), paired Student's t test (D, Z+, t(8) = 3.14, p = 0.014, n = 9; Z-, t(8) = 0.183, p = 0.859, n = 9; E, Z+, t(8) = -2.13, p = 0.065, n = 9; Z-, t(8) = -0.425, p = 0.682, n = 9; J, Z+, t(8) = 2.074, n = 9, p = 0.072; Z-, t(8) = 1.690, p = 0.151, n = 9; K, Z+, t(8) = -3.942, p = 0.004, n = 9; Z-, t(8) = 1.845, p = 0.102, n = 9), and unpaired Student's t test (C, control Z+/Z-, t(24) = -2.33, p = 0.029, n13, 13; apamin Z+/Z-, t(16) = 3.56, p = 0.003, n = 9, 9; Z+ control/apamin, t(20) = 1.19, p = 0.248, n = 13, 9; Z-control/apamin, t(20) = -8.46, p = 0.0000, n = 13, 9; D, baseline Z+/Z-, t(16) = 0.520, p = 0.610, n = 9, 9; E, baseline Z+/Z-, t(16) = 1.410, p = 0.178, n = 9, 9; F, fAHP/Z+, t(20) = 1.924, p = 0.068, n = 13, 9, fAHP/Z-, t(20) = -0.418, p = 0.681, n = 13, 9, mAHP/Z+, t(20) = -0.331, p = 0.744, n = 13, 9, mAHP/Z-, t(20) = -2.51, p = 0.021, n = 13, 9; I, iberiotoxin Z+/Z-, t(16) = -2.76, p = 0.014, n = 9, 9; Z+ control/iberiotoxin, t(20) = 1.45, p = 0.164, n = 13, 9; Z-control/iberiotoxin, t(20) = 0.350, p = 0.730, n = 13, 9; J, baseline Z+/Z-, t(16) = 1.095, p = 0.290, n = 9, 9; K, baseline Z+/Z-, t(16) = -1.013, p = 0.326, n = 9, 9; L, fAHP/Z+, t(20) = -0.249, p = 0.806, n = 13, 9, fAHP/Z-, t(20) = 1.044, p = 0.309, n = 13, 9, mAHP/Z+, t(20) = -0.034, p = 0.973, n = 13, 9, mAHP/Z-, t(20) = -0.415, p = 0.682, n = 13, 9). ∗∗∗p < 0.001, ∗∗p < 0.01, ∗p < 0.05. All recorded PCs were located in zones 1+, 1-, and 2+ in lobule IV-V.

Under application of apamin, higher spike counts were evoked at 300–500 nA current injections in both Z+ and Z- PCs than under the control condition without apamin (Figure 4B). Tetanization (LTP-IE protocol) produced significant intrinsic plasticity, i.e., increase in intrinsic excitability, in Z+ PCs, which lasted 20 min after the tetanization (Figure 4B left, F(1,96) = 9.00, p = 0.003, two-way ANOVA with repeated measure, n = 9). In comparison, apamin completely blocked intrinsic plasticity in Z- PCs (Figure 4A, bottom, and 4B right). The degree of intrinsic plasticity was compared between control and apamin conditions by plotting the change of the spike count evoked by 300 pA current injection (Figure 4C). Although apamin did not much affect the spike count change in Z+ PCs (Figure 4C left), apamin made the intrinsic plasticity significantly smaller, and nearly completely occluded, in Z- PCs (Figure 4C, Z+ control/apamin, t(20) = 1.19, p = 0.248, n = 13, 9; Z-control/apamin, t(20) = -8.46, p = 0.0000, n = 13 and 9 PCs).

Then we examined the effect of apamin on the change of AHPs produced by the LTP-IE protocol. As for the fAHP, a significant decrease was observed in Z + PCs (Figure 4D left, t(8) = 3.14, p = 0.014, paired t test, n = 9) but no significant change was observed in Z- PCs (Figure 4D right, t(8) = 0.183, p = 0.859, n = 9). These changes were similar to those observed in the control condition without apamin (gray back lines in Figure 4D). As for the mAHP, the apparent increase was not significant in Z+ PCs (Figure 4E left, t(8) = -2.13, p = 0.065, paired t test, n = 9) and no change was observed in Z- PCs (Figure 4E right, t(8) = -0.425, p = 0.682, n = 9). It indicated that the mAHP increase in Z+ PCs and the mAHP decrease in Z- PCs observed in the control condition (gray back lines in Figure 4E) both disappeared under apamin. No significant changes in sAHP were observed in both Z+ and Z- PCs (Figure S4C), akin to the control condition. We further compared these tetanization-dependent changes of AHPs with those under the control condition (Figure 4F). Apamin affected (suppressed) only the tetanization-dependent change (decrease) of mAHP in Z- PCs (Figure 4F). Thus, suppressing the tetanization-dependent decrease of mAHP was most likely correlated with the occlusion of intrinsic plasticity in Z- PCs under apamin.

We also similarly examined the effects of iberiotoxin. Under the application of iberiotoxin, both Z+ and Z- PCs showed intrinsic plasticity or a significant increase in the spike count-current injection relationship (Figures 4G and 4H) as in the control condition. Five Z+ PCs out of nine showed almost no spike count changes (0%–9.5%), whereas four Z+ PCs and all nine Z- PCs showed some spike count changes (22.2%–80.0%), by the LTP-IE protocol under iberiotoxin. However, at the level of the entire population, no significant effects were observed in the comparison of intrinsic plasticity between control and iberiotoxin conditions, as represented by the change in the spike count evoked by 300 pA, in Z+ PCs (Figure 4I, Z+ control/iberiotoxin, t(20) = 1.45, p = 0.164, n = 13, 9) or in Z- PCs (Figure 4I, Z- control/iberiotoxin, t(20) = 0.350, p = 0.730, n = 13, 9). In measurements of AHPs, the LTP-IE protocol produced an increase of mAHP in Z+ PCs (Figure 4K, t(8) = -3.942, p = 0.004, paired t test, n = 9), which was similar to the change in the control condition, but no significant changes in other conditions (Figures 4J and 4K). In comparing tetanization-dependent changes of AHPs between the control and iberiotoxin conditions, no significant effects were observed by the application of iberiotoxin in fAHP, mAHP, or sAHP in either Z+ or Z- PCs (Figures 4L and S4D).

These results indicated that different expressions or controls of calcium-activated potassium channels underlie the different responses of the intrinsic plasticity change to the LTP-IE protocol between Z+ and Z- PCs. Specifically, apamin-sensitive SK2 channels appeared to play a fundamental role in inducing the LTP-IE by decreasing mAHP in Z- PCs. In contrast, iberiotoxin-sensitive BK channels seemed to have only limited involvement in intrinsic excitability or plasticity in Z+ and Z- PCs. The possibility that these channels may be more involved in a subpopulation of Z + PCs, as suggested by the iberiotoxin block of intrinsic plasticity (above), than in other PCs was not further examined.

### Different degrees of PF-PC synaptic LTP between Z+ and Z- PCs

The intrinsic plasticity shares the same molecular pathway as the postsynaptic molecular pathway of the LTP of the PF-PC synaptic transmission (Belmeguenai et al., 2010). Therefore, to further elucidate intrinsic plasticity pathways in Z+ and Z- PCs, we compared the LTP of the PF-PC synaptic transmission between Z+ and Z- PCs. We continuously monitored the excitatory postsynaptic current (EPSC) at the PF-PC synapse and stimulated the PF every 1 min under voltage-clamp mode in Z+ and Z- PCs. In the middle of monitoring, PF stimulation frequency was increased to 1 Hz for 5 min to induce synaptic LTP. Although both presynaptic and postsynaptic mechanisms are involved in the LTP of the PF-PC synaptic transmission (Bouvier et al., 2016; Wang et al., 2014), our method was focused more on the postsynaptic mechanisms based on the following. First, parasagittal slices, which we used, are supposed to better bypass the involvement of the presynaptic NMDA receptor in PFs, presumably by the closeness of the stimulation (Bouvier et al., 2016). Second, the low-frequency stimulation of PFs is supposed to reduce the presynaptic nitrogen monoxide release (Wang et al., 2014).

**Figure 5:**
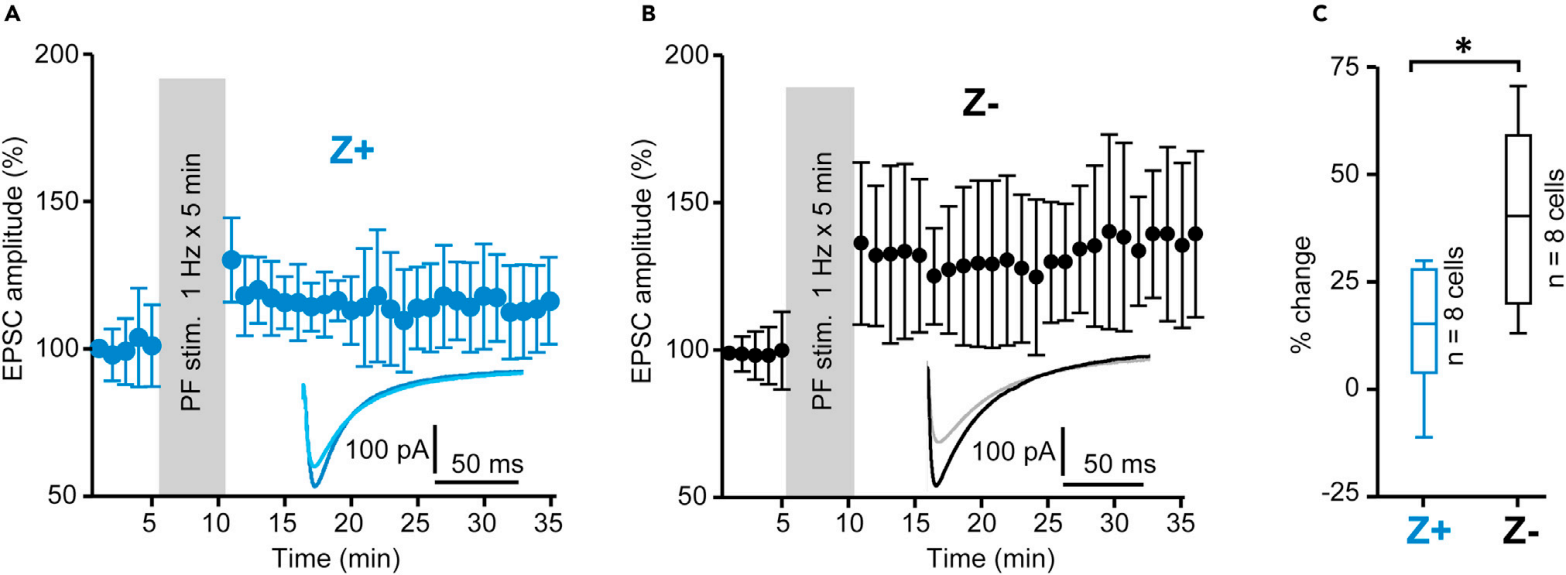
Comparison of the LTP in the PF-PC synaptic transmission between Z+ and Z- PCs in neighboring zones (A and B) Time graph of the normalized EPSC amplitude before and after the 1-Hz PF stimulation in eight Z+ PCs in zebrin zone 1+ and 2+ (A) and in eight Z- PCs in zebrin zone 1- (B) in lobule IV-V. (C) Comparison of the EPSC amplitude 21–25 min after the 1-Hz PF stimulation. Data are represented as mean ± standard deviation in (A and B) and Tukey method box and whisker graphs in (C). The significant difference was tested with unpaired Student's t test (t(14) = -2.69, p = 0.017, n = 8, 8). ∗p < 0.05.

The PF stimulation at 1 Hz for 5 min produced synaptic LTP in Z+ and Z- PCs (Figures 5A and 5B). Comparison of the EPSC amplitudes before and 21–25 min after the 1-Hz PF stimulation produced significant difference with paired Student's t test in both Z+ (t(7) = 2.553, p = 0.038, unpaired t test, n = 8) and Z- (t(7) = 4.860, p = 0.0018, n = 8) PCs. In addition, the potentiation percentage, i.e., the ratio of the average EPSC 20–25 min after the stimulation to the average EPSC before the stimulation, was significantly higher in Z- PCs than in Z+ PCs (Figure 5C, t(14) = -2.69, p = 0.017, Student's t test, n = 8). Thus, the results showed that the PF-PC synaptic transmission LTP was more enhanced in Z- PCs than in Z+ PCs.

## Discussion

The present study compared intrinsic synaptic properties between Z+ and Z- PCs located in neighboring zebrin zones in the slice preparation in AldocV mice. Z- PCs showed stronger intrinsic excitability, intrinsic plasticity, and PF-PC synaptic LTP than Z+ PCs. The involvement of SK channels in intrinsic plasticity was significant only in Z- PCs. As a whole, the present study demonstrated heterogeneous cellular physiological properties linked to the heterogeneous molecular expression in PCs located in neighboring zebrin zones.

### Electrophysiological heterogeneity in PC populations

Since the discovery of the zonal distribution of PC populations heterogeneous in expression levels of some molecules, it has been a long-lasting question whether such PCs have different physiological properties that affect the cerebellar function. In the present study, we sampled Z+ and Z- PCs from identified neighboring zones in specific lobules in the slice preparation from P16-24 mice with fluorescent labeling of specific populations of PCs. By eliminating possible effects caused by the differences in lobule-related factors, afferent inputs, and inhibitory interneuron inputs, which were not necessarily eliminated in previous *in vivo* and *in vitro* studies, we could focus on different cellular physiological properties between Z+ and Z- PCs. Our initial study in AldocV mice has shown no difference in input resistance, capacitance, or basic properties in PF-PC synaptic transmission between Z+ and Z- PCs in vermal lobule VIII (Nguyen-Minh et al., 2019). However, this study detected some differences in intrinsic excitability between these PCs, which led us to the present study.

PCs are capable of firing simple spikes repetitively. Sampling PCs in various cerebellar areas in *in vivo* preparation showed that Z- PCs generally show a higher frequency of simple spike firing (Xiao et al., 2014; Zhou et al., 2014). The results of the present study showed that the difference in PC intrinsic excitability underlies the different firing frequencies between Z+ and Z- PCs. Since Z+ and Z- PCs have similar size, input resistance, and capacitance, ionic mechanisms activated by depolarization directly or indirectly for the difference of the intrinsic excitability would be responsible for this difference. The results indicated that apamin-sensitive SK channels were involved in controlling the spike firing rate in both Z+ and Z- PCs; it was not determined whether they are responsible for the different firing rates between Z+ and Z- PCs. Wu et al. (2019) reported that transient receptor potential cation channel C3 (TRPC3), which is more highly expressed in Z- PCs than in Z+ PCs in the vermis, is responsible for the stronger intrinsic excitability of Z- PCs. In the present study, stronger intrinsic excitability was seen in the vermis and the hemispheric area in Crus II (zones 5+ and 5-). Since TRPC3 is similarly highly expressed in Z+ and Z- PCs in the hemispheric area (Wu et al., 2019), other ionic channels must also be involved in tuning different intrinsic excitability between Z+ and Z- PCs. The present study suggested that the lower activity of some K+ channels activated directly or indirectly (through Ca^2+^ influx) by depolarization, which generates the smaller AHP and is related to the higher intrinsic excitability in Z- PCs. A linked decrease of AHP and increase of intrinsic excitability occurs by a reduction of K^+^ conductance in many types of neuronal plasticity (Abraham, 2008; Belmeguenai et al., 2010; Disterhoft and Oh, 2006).

New findings of this study include that Z- PCs maintain input-output translation sensitivity at stronger current injection ranges than Z+ PCs. More importantly, tetanization enhances intrinsic excitability more in Z- PCs than in Z+ PCs, evidencing higher intrinsic plasticity in Z- PCs. The underlying mechanisms for the intrinsic plasticity were different between Z- and Z+ PCs; a decrease in mAHP occurred in Z- PCs, whereas a decrease in the fAHP occurred in Z+ PCs. Effects of the SK channel blocker, apamin, on the spike-current relationship and the AHP were also quite different between Z+ and Z- PCs. It has been shown that SK2 channels play the leading role in producing intrinsic plasticity in PCs (Belmeguenai et al., 2010; Grasselli et al., 2016). Although these studies did not specify zebrin types of recorded Purkinje cells, it is likely that their recorded PCs were mostly Z- since lobule IV-V, in which Z- zones are much wider than Z+ zones, have been usually used in *in vitro* and *in vivo* studies. Findings in Z- PCs in the present study generally agree with the results of these studies. However, completely different results indicating absence of apamin effects on intrinsic plasticity were obtained from Z+ PCs in the present study. The results support the heterogeneous cellular physiological properties between Z+ and Z- PCs.

Besides intrinsic plasticity, it was shown in the present study that the LTP of the PF-PC synaptic transmission was stronger in Z- PCs than in Z+ PCs. Z- PCs also show stronger long-term depression (LTD) than Z+ PCs (Wadiche and Jahr, 2005). As a whole, Z- PCs are more sensitive to tonic excitatory synaptic input and respond to a higher frequency of firing and are more sensitive to the enhancement of simple spike activity than Z+ PCs.

PCs in vermal lobules III and IV-V are generally used to research synaptic transmission in the cerebellar slice preparation. Since Z- PCs are much more abundant than Z+ PCs in these lobules, it would be needed to consider the possibility that reported findings on the PC synaptic and intrinsic properties, including the robustness of the Ca^2+^-dependent modulation of the LTP and LTD threshold (Piochon et al., 2016), were based mainly in Z- PCs. Therefore, based on present study results, we like to claim the general importance of specifying localization and zebrin types of PCs in *in vitro* and *in vivo* studies.

The depolarization block in the spike-current relationship observed in PCs, in Z+ PCs in particular, in the present study was stronger than that reported in previous studies (Shim et al., 2018; Zhou et al., 2014). This may be because the data in these previous studies were obtained presumably mostly from Z- PCs. However, the temperature effect may also be possibly involved since those previous studies and the present study were performed at 30°C–34°C and at room temperature, respectively.

### Functional significance of the electrophysiological heterogeneity in cerebellar zones

Z- and Z+ PCs are distributed separately in tens of longitudinal zones in the cerebellar cortex. Each zone has a topographic connection with particular subareas of the inferior olive through climbing fibers and particular subareas of the cerebellar nucleus through PC axons (Pijpers et al., 2006; Sugihara and Shinoda, 2004). Zones of Z+ PCs (or Z+ zones) tend to receive climbing fiber input from regions of the inferior olive dominated by descending inputs, whereas Z- zones receive climbing fiber input from regions of the inferior olive that receive predominantly peripheral inputs (Cerminara et al., 2015; Sugihara and Shinoda, 2004; Voogd and Ruigrok., 2004). Indeed, a Ca^2+^ imaging study reported CF-dependent activity at different phases in Z+ and Z- zones in crus II, in which both Z+ and Z- zones occupy comparable widths in behaving mice (Tsutsumi et al., 2015, 2019).

So far, the functional significance of different intrinsic and synaptic properties between Z+ and Z-PCs has been considered in relation to the lobular functional localization. Cortical areas that control somatosensory reflexes such as eye blinking reflexes (hemispheric lobule V and simple lobule) and locomotion (vermal lobules I-VIa) are mostly composed of wide Z- zones and narrow Z+ zones. In contrast, cortical areas that control eye movements (flocculus and nodulus), posture, and cognitive function (crus I and vermal lobules VI-VII) are mainly composed of only wide Z+ zones. Thus, different types of PC excitability and plasticity may be well tuned for different types of learning and adaptation mediated in these areas. Indeed, there is a proposal of such an idea in adaptation mechanisms of eye-blink response and vestibule-ocular response (De Zeeuw and Ten Brinke, 2015). The quick and robust plasticity in Z- PC supports the "temporal coding" mechanism (the spikes occur at millisecond precision) of eye-blink conditioning between unrelated unconditioned and conditioning stimuli. The small plasticity in Z+ may fit with "rate coding" in the vestibulo-ocular reflex adaptation that requires precise adjustment of correlated movements between the head and eye (De Zeeuw and Ten Brinke, 2015). The present results support these ideas.

Furthermore, clear functional differences in PCs located in neighboring Z+ and Z- stripes demonstrated in the present study in lobule IV-V and crus II would propose functional contrast among zebrin zones within each lobule. All cerebellar lobules contain Z+ and Z- zones, although their widths vary (Fujita et al., 2014). Furthermore, the expression levels of zebrin (aldolase C) are not necessarily constant but variable to some extent in each Z+ and Z- zone in some lobules. Therefore, we propose that PCs' different intrinsic and synaptic properties related to the zebrin type may be tuned distinctly in each zebrin zone in each lobule throughout the cerebellum.

### Molecular mechanisms underlying the electrophysiological heterogeneity

**Figure 6:**
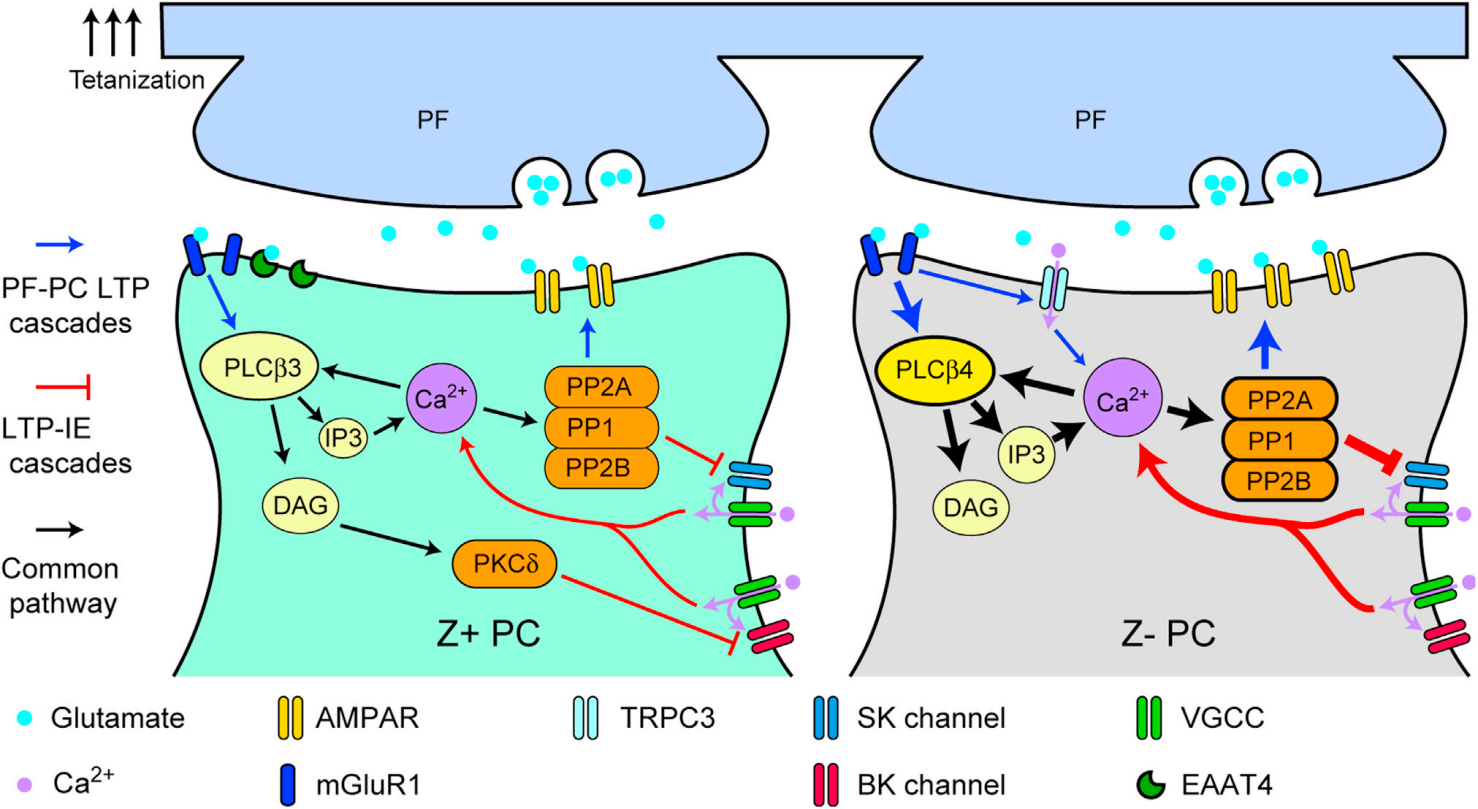
Scheme of putative molecular mechanisms underlying synaptic and intrinsic plasticity in Z+ and Z- PCs Pathways involved in the LTP of the PF-PC synapse are colored in blue, whereas pathways involved in intrinsic plasticity, not necessarily localized in the spine, are colored in red. Common pathways are in black. Refer to the last section of the discussion for an explanation. Abbreviations: PF, parallel fiber; PC, Purkinje cell; AMPAR, AMPA receptor; PKC, protein kinase C; PLC, phospholipase C; PP1, protein phosphatase 1; PP2A, protein phosphatase 2A; PP2B, protein phosphatase 2B; SK, small conductance calcium-activated potassium channel; BK, large-conductance maxi calcium-activated potassium channel; PKCδ, protein kinase C delta type; DAG, diacylglycerol; LTP: Long-term potentiation; LTP-IE: long-term potentiation of intrinsic excitability.

Many molecules involved in controlling neuronal excitability and synaptic transmission have heterogeneous expression profiles linked to the zebrin expression patterns Cerminara et al. (2015); Hawkes (2014): Z+ PCs preferentially express PKCδ, EAAT4, and PLCβ3, whereas Z- PCs preferentially express PLCβ4 and TRPC3 (only in the vermis, Wu et al., 2019). These differences in molecular expression may be related to the different electrophysiological properties between Z+ and Z- PCs observed in the present study (Figure 6). We propose that the following mechanism supports enhanced LTP in the PF-PC synaptic transmission in Z- PCs. In the tetanic PF stimulation for the LTP of PF-PC synaptic transmission, the lack of EAAT4 in Z- PCs induces higher activation of mGluR1 in the extrasynaptic area in Z- PCs (Wadiche and Jahr, 2005). The mGluR1 activation, through the mediation of Gq alpha subunit, induces PLCβ4 activation, which then induces inositol-1,4,5-triphosphate (IP3)-dependent Ca^2+^ release from the intracellular Ca^2+^ storage. Interaction of the mGluR1b receptor with the Ca^2+^-permeable TRPC3 channel (Wu et al., 2019) is another possible pathway for Ca^2+^ increase in Z- PCs. The LTP-IE protocol can induce increased intracellular Ca^2+^ concentration in Z- PCs because of a larger influx through voltage-activated Ca^2+^ channels due to more frequent action potentials in Z- PCs. Ca^2+^ then activates PLCβ4 further and also protein phosphatase 1 (PP1), protein phosphatase 2A (PP2A), and protein phosphatase 2B (PP2B), which induce LTP of the PF-PC synaptic transmission (Belmeguenai et al., 2010). Furthermore, these phosphatases also suppress the small conductance Ca^2+^-activated K channel (SK channel) putatively involved in the mAHP (Belmeguenai et al., 2010; Grasselli et al., 2020; Titley et al., 2020). The present result showed specific involvement of the SK channel in the LTP-IE of Z- PCs, thereby suggesting that the blockade of the SK channel by these phosphatases activated by Ca^2+^ is the mechanism of the enhanced LTP-IE in Z- PCs. On the contrary, in Z+ PC, PLCβ3-induced diacylglycerol (DAG) release can activate PKCδ, which requires DAG but not Ca^2+^ for activation (Rosse, et al., 2010). A decrease of PKCδ expression occurs in lobule X (entirely Z+) PCs during cerebellar adaptation after unilateral labyrinthectomy (Barmack et al., 2001). Involvement of PKCδ in PC plasticity may be mediated by the modulation of large-conductance Ca^2+^-activated K (BK) channels as indicated in Figure 6 (Widmer et al., 2003). Although the general negative effect of iberiotoxin (50 nM, BK channel blocker) on the intrinsic plasticity and AHP amplitude in Z+ PCs, as well as in Z- PCs, did not simply support this idea, BK channels may possibly be involved in a subpopulation of Z+ PCs that showed no spike count change by LTP-IE protocol under iberiotoxin (see results in relation to Figures 4H and 4I). As a whole, the heterogeneous electrophysiological properties observed in the present study between Z- and Z+ PCs suggest that differences in the molecular expression profiles are involved in fundamental functional differences between these PCs.

### Limitations of the study

The cerebellar cortex is separated into more than 20 zebrin zones (Fujita et al., 2014), and the patterns of zebrin zones are distinct in different lobules. It is not practical to study the property of Purkinje cells in many different zebrin zones. Therefore, we mainly focused on particular neighboring zebrin stripes (zones 1+, medial 1-, lateral 1-, and 2+ in lobule IV-V). Although we obtained similar results in zebrin zones in different areas (zones 5+ and 5- in crus II, as shown in supplemental information), PCs in other areas were not studied. They are to be studied in the future. This study demonstrated the involvement of SK and BK channels in the different intrinsic plasticity with a pharmacological approach. Different sophisticated approaches (e.g., genetic) would unravel details of cellular and molecular basis that underlie the link between the cellular physiology and cortical zones in the cerebellum.

The present slice patch-clamp experiments were performed at room temperature. We cannot exclude the possibility that the temperature effect affected the results, the depolarization block in the spike-current relationship, for example.

## STAR★Methods

### Key resources table

**Table:** 

REAGENT or RESOURCE	SOURCE	IDENTIFIER
**Antibodies**		

Rabbit anti-aldolase C (Aldoc)	Dr. Izumi Sugihara (Tokyo Medical and Dental University, Tokyo, Japan; Sugihara and Shinoda, 2004), rabbit polyclonal, Immunogen: Synthetic peptide conjugated to KLH derived from the amino acids 322-344 of rat aldolase C: CGAATEEFIKRAEMNGLAAQGKYE	Lot # 69076, RRID:AB_2313920
Donkey anti-rabbit IgG alexa fluor 594	Jackson ImmunoResearch	Cat# 711-585-152

**Experimental models: Organisms/strains**		

Mouse: AldocV, aldoc-Venus: C57BL/6N strain	Comprehensive Brain Science Network	MGI:5620954, Fujita, et al., 2014
Mouse: Rgs8-EGFP, Tg(Rgs8-EGFP)CB132Gsat:C57BL/6N	GENSAT Project	MGI:3844513, http://www.informatics.jax.org/allele/MGI:3844513
Mouse: C57BL/6N	CLEA Japan	N/A

**Chemicals**		

Iberiotoxin Apamin	Almone labs Abcam	STI-400 Ab120268

**Software and algorithms**		

pClamp 10, Clampex, Clampfit	Axon Instruments / Molecular Devices, San Jose, CA, USA	N/A
Excel	Microsoft	RRID: SCR_016137
Zen	Zeiss, Oberkochen, Germany	RRID: SCR_013672
Adobe photoshop 7	Adobe, San Jose, CA, USA	N/A
Adobe illustrator 10.3	Adobe	N/A
R	The R Foundation	https://www.r-project.org

**Deposited data**		

The Excel file that contains all original data of experiments and statistics details of this paper	Mendeley Data site, uploaded by Dr. Izumi Sugihara	Mendeley Data: https://doi.org/10.17632/bm2gk232mt.1

### Resource availability

#### Lead contact

Requests for resources, datasets, protocols, and any other additional information should be directed to and will be fulfilled by the Lead Contact, Izumi Sugihara (isugihara.phy1@tmd.ac.jp).

#### Material availability

This study did not generate new unique reagents.

### Experimental model and subject details

#### Animals

Experimental protocols were approved by the Animal Care and Use Committee (A2021-065A, A2019-187C3, A2018-148A) and Gene Recombination Experiment Safety Committee (G2019-020C5) of Tokyo Medical and Dental University. Heterozygous mice (N = 78) of both sexes at postnatal day 16–24 (P16–24) of the Aldoc-Venus knock-in line (AldocV, Fujita et al., 2014) with C57BL/6N background were used. We consistently use this young age of mice in all of our experiments. In AldocV mice, aldolase C (zebrin II)-positive PCs (Z+ PCs) are labeled by the mutated green fluorescent protein, Venus (Fujita et al., 2014).

Mice were bred and reared in a specific pathogen-free, 12-12-hour light-dark cycled condition with freely available food and water in the animal facility of the university. Heterozygous Aldoc-Venus pups were obtained by mating male homozygous Aldoc-Venus mice (Fujita et al., 2014; Sarpong et at., 2018) and wild-type C57BL/6N females.

Some experiments (shown in Figure 2) were done in heterozygous Rgs8-EGFP mice (N = 8, both sexes at P16–24). The Rgs8-EGFP mouse is a transgenic reporter line with the transgene containing the coding sequence of enhanced green fluorescent protein (EGFP) inserted into the mouse genomic bacterial artificial chromosome (BAC) at the transcription initiation codon of the regulator of G-protein signaling 8 (Rgs8) gene. They show fluorescent protein expression in zebrin-negative PCs. Our systematic analysis of the expression pattern of these mice will be published later. PCR genotyping was performed using tail genomic DNA with two primers (Rgs8-108F, TTTAGGTGAGAGGACGTGAGAG; GFP-R, GCGGTCACGAACTCCAGC), which corresponded to the upstream sequence of the GFP insertion site and the coding sequence of the EGFP cDNA of the RGS8-EGFP transgene, respectively (PCR product size, 788 bp; Kaneko et al., 2018). We established and maintained a homozygous Rgs8-EGFP mouse colony. Heterozygous Rgs8-EGFP pups were obtained by mating homozygous Rgs8-EGFP males and wild-type C57BL/6N females.

### Method details

#### Slice preparation

Mice of both sexes at postnatal day 16–24 (P16–24) were anesthetized with an intraperitoneal injection of an overdose of pentobarbital (0.1 mg/g, Abbott lab, Chicago, U.S.A.) and xylazine (0.005 mg/g) and euthanized by cervical dislocation. Firstly was the dissection of the cerebellar block from the extracted brain under ice-cold sucrose cutting solution containing (in mM): 87 NaCl, 2.5 KCl, 0.5 CaCl_2_, 7 MgCl_2_, 1.25 NaH_2_PO_4_ ·2H_2_O, 10 D-glucose, 25 NaHCO_3_, 75 Sucrose and saturated with 95% O_2_ and 5% CO_2_. Next, 200–300 μm parasagittal slices were cut using a vibratome (PRO7, Dosaka, Osaka, Japan). Slices were initially allowed to recover in artificial cerebrospinal fluid (ACSF) solution containing (in mM): 128 NaCl, 2.5 KCl, 2 CaCl_2_, 1 MgCl_2_, 1.2 NaH_2_PO_4_, 26 NaHCO_3_, and 11 glucose and saturated with 95% O_2_ and 5% CO_2_ at 34°C for 30 min, and then allowed to recover in ACSF at room temperature for at least 1 hour.

#### Zebrin stripe identification in slices

In the sagittal sections used in experiments of the present study, it was not straightforward to identify zebrin zones. Zebrin zones are not entirely parallel to the midsagittal plane. Instead, they are tilted laterally in the dorsal position and shifted laterally in the central part of the cerebellum. Furthermore, individual zebrin zones have different widths. For example, zone 1+ ran nearly in parallel with the plane of the slice throughout lobule IV-V in the slice at the midsagittal section. Zones 2+ ran more laterally in the caudal part and more medially in the rostral part of lobule IV-V in the section approximately 400–500 μm from 1+. When cutting sagittal slices, we kept them orderly to easily track the mediolateral position of each slice. Because of these morphological properties, roughly shown in the unfolded scheme (Figure 1C, Fujita et al., 2014), zebrin stripes appeared in a characteristic pattern in sagittal sections. Therefore, we could record from Z+ and Z- PCs in identified zebrin zones (1+, 1-, 2+ in lobule IV-V of Aldoc-Venus mice, Figures 1A and 1B)

#### Patch-clamp recordings

The slices were examined by placing them in the bottom of the recording chamber soaked in ACSF, then mounted on a microscope stage (BX51IW, Olympus, Tokyo, Japan), and visualized by using 10× objective and epifluorescence optics with a filter for appropriate wavelength selection. We carefully checked the distance of the sliced plane from the midline, location, and tilt of Z+ zones. After identifying Z+ and Z- zones in lobule IV-V in the slice (above), approached the apex or wall of lobule IV-V with the patch electrode. The objective of the microscope was switched to a 40× water immersion. The microscope's optics were changed accordingly between epifluorescence and near-infrared Nomarski differential interference-contrast (IR-DIC) systems to approach the PC with the electrode. PCs were visualized for recording using a 40× water-immersion objective.

Slices were superfused constantly with ACSF at room temperature (24°C). 100 μM picrotoxin (C0375, Tokyo Chemical Industry Co., Tokyo, Japan) was added to the ACSF to block GABAA channels. Patch electrodes (3–5 MΩ) were pulled with a Flaming/Brown type micropipette puller (P-97, Sutter Instruments, San Jose, CA, USA) and filled with the internal solution consisting of the following (in mM): 124 K-gluconate, 2 KCl, 9 HEPES, 2 MgCl_2_, 2 MgATP, 0.5 NaGTP, 3 L-Ascorbic Acid, pH adjusted to 7.3 with KOH, osmolarity was adjusted to 280–300 mOsm with sucrose. Signals from the patch pipette were recorded with a MultiClamp 700B amplifier (Molecular Devices, San Jose, USA), digitized at 10–20 kHz, filtered at 2–5 kHz with a Digidata 1440A analog-to-digital converter (Molecular Devices) and stored under the control with Clampex 10.7 software (Molecular Devices).

The stimulation electrode was made from a glass pipette (0.5–1 MΩ) filled with ACSF and a pair of stainless wires placed inside and outside of the pipette. It was positioned in the molecular layer to stimulate parallel fibers (PFs). In all experiments, the capacitance was compensated, the impedance bridge was balanced, and bias current (<400 pA) was injected to keep the membrane voltage between −65 and −70 mV. Access resistance was <20 MΩ, and both access and input resistances were monitored by applying hyperpolarizing voltage steps (−10 mV) in voltage-clamp mode and −300 pA in current-clamp mode at the end of each sweep, and changed by <20% throughout the recording.

The whole-cell patch-clamp recording was made from randomly selected Z+ and Z- PCs in identified zebrin zones in the apex and folial wall of lobule IV-V and crus II. All the experiments were done in current-clamp mode, except for the synaptic LTP experiments, which were done in voltage-clamp mode. Holding potential was set at −70 mV with a negative holding current of <400 pA in recordings in voltage- and current-clamp modes.

#### Measurements of intrinsic excitability and intrinsic plasticity of PCs

We evaluated PCs' excitability by a series of 500-ms long square-shaped current steps of intensity ranging from 0 pA to 1000 pA or 500 pA with increments of 50 pA or 100 pA injected at 7-second intervals from the baseline membrane potential of 70 mV under current-clamp mode. We measured the number of action potentials during the 500 ms period (spike count, Figures 1D and 1F; row data in Figure S1A) and the time between the first and the last spike, defined by the amplitude larger than 5 mV (firing duration, Figure S1C and S1D) for each current injection to obtain the current-spike relationship (Figure 1). The current intensity which elicited the maximum number of spikes was also obtained (“maximum firing current,” Figure S1B).

We gave tetanizing stimulation to induce intrinsic plasticity or long-term potentiation of intrinsic stimulation (LTP-IE). A set of tetanizing stimulation consisted of 150 repetitions (every 2 s for 5 min) of a run of five suprathreshold square-shaped depolarizing current injections (400 pA, 100 ms ON and OFF, 5 Hz for 1 s, as shown in Figure 3A) under current-clamp mode (“long-term potentiation of intrinsic stimulation, LTP-IE protocol”, Shim et al., 2017). We measured the current-spike relationship (above) immediately before the LTP-IE protocol (“baseline”) and 10 and 20 min after the LTP-IE protocol (“10 min”, and “20 min”, Figure 3C). In control experiments, we measured the current-spike relationship at the same timing without giving the LTP-IE protocol.

#### Measurements of subthreshold afterhyperpolarization (AHP) of PCs

The size of the subthreshold AHP was measured in two ways from the baseline membrane potential of 70 mV under current-clamp mode. First, in the synaptic protocol, PFs were stimulated with a train of 5 stimuli of 100 Hz to elicit a summed EPSP of 5–12 mV in a PC, which was followed by a subthreshold AHP of 0.5–3 mV (Figure 1H). The peak amplitude of the subthreshold AHP was divided by the peak amplitude of the summed EPSP to obtain “AHP/EPSP ratio” (Figure 1I). Second, in Rgs8-EGFP mice, 50 pA current injection was given through the patch pipette for 500 ms to produce a subthreshold AHP after the cease of the current injection. The peak amplitude of the AHP was measured (Figure 2E).

#### Measurements of distinct components of afterhyperpolarization (AHP) of PCs

Distinct components (fAHP, mAHP, and sAHP) were measured during and after spike responses evoked by 500-ms long square-shaped current steps under current-clamp mode. The fast AHP (fAHP) was measured in the AHP at the first action potential evoked by the smallest square current injection among six steps (0 pA to 500 pA, 100 pA increment) which evoked an action potential (usually 100 pA). Subsequently, the amplitude of the voltage difference between the negative peak (bottom) of the AHP and the threshold level or the deflection point of the membrane potential (Figure 1D top) was measured.

To measure the medium AHP (mAHP) and slow AHP (sAHP), a burst of action potentials were evoked in a PC by a 300 pA square current injection under current-clamp mode. The cease of the current injection produced an elongated AHP, which peaked in about 100–200 ms and lasted for about 1 sec. The mAHP and sAHP were obtained from the voltage difference between the baseline and the negative peak of the elongated AHP and between the baseline and the decaying AHP 500 ms after the negative peak, respectively (Figure 3D bottom and Figure S4A).

#### Measurements of the long term potentiation (LTP) of the parallel fiber (PF)-PC synaptic transmission

Intensity of the PF stimulation was adjusted to produce EPSC of 200–300 pA. The amplitude of the EPSC in response to the parallel fiber stimulation given every 20s (0.05 Hz) was monitored under voltage-clamp mode and recorded with the online statistic of Clampex. Five minutes after the start of monitoring, recording was swithced to current-clamp mode and the PF stimulation frequency was switched to 1 Hz (LTP induction protocol with the low-frequency PF stimulation) for 5 minutes. Then, the EPSC response to the 0.05 Hz PF stimulation was monitored under voltage-clamp mode for 25 minutes (Figure 5). Throughout the synaptic LTP experiments (Figure S5), series resistance of the patch pipette was also monitored.

#### Pharmacology experiments

In pharmacology experiments, 50 nM iberiotoxin (Alomone Labs, STI-400) or 100 nM apamin (Abcam, ab120268) was added into the ACSF. Then, intrinsic excitability, intrinsic plasticity, and distinct components of AHP were measured in PCs as above.

#### Histological procedures (for Rgs8-EGFP mice)

Heterozygous female Rgs8-EGFP pup (N = 1) at P20 were anesthetized with an intramuscular injection of pentobarbital sodium (0.1 mg/g body weight) and xylazine (0.005 mg/g body weight) and perfused transcardially with phosphate-buffered saline (PBS, pH 7.4) with heparin sulfate (0.1%), and then with 4% paraformaldehyde. The brains were dissected in chilled paraformaldehyde, kept in 4% paraformaldehyde for post-fixation, and then soaked in sucrose solution (30% with 0.05 M phosphate buffer, pH 7.4) for two days. Brains were then coated with gelatin solution (10% gelatin, 10% sucrose in 10 mM phosphate buffer, pH 7.4, 32°C). The gelatin block was hardened by chilling and then soaked overnight in a fixative with a high sucrose content (4% paraformaldehyde, 30% sucrose in 0.05 M phosphate buffer, pH 7.4). Serial sections were cut coronally and sagittally using a freezing microtome at a thickness of 50 μm. Some sections were mounted on the glass slides, while other sections were rendered to immunostaining for aldolase C. After washing with PBS, floating sections were incubated with the primary rabbit antibody against aldolase C (Key Resources Table; concentration, 1:10000) for two days. After washing with PBS, they were incubated overnight with the secondary antibody conjugated with Aleda Fluor 594 (Key Resources Table; concentration, 1:400). Sections were then mounted on glass slides. Mounted sections were dried and coverslipped with a water-soluble mounting medium (CC mount, Sigma C9368-30 ML).

Fluorescence images were digitized using a cooled color CCD camera (AxioCam ICm1, Zeiss, Oberkochen, Germany) attached to a fluorescent microscope (AxioImager.Z2, Zeiss) in 12-bit gray-scale with an appropriate filter set. The cerebellum sections were digitized using the microscope's 2.5× objective and tiling function to control digitizing (Zen 2 Pro, Zeiss). Micrographs were adjusted concerning contrast and brightness and assembled using software (ZEN 2 Pro, Zeiss and Photoshop 7, Adobe, San Jose, CA, USA). An appropriate combination of pseudo-color was applied to fluorescent images. Photographs were assembled using Photoshop and Illustrator software (Adobe). The software was used to adjust contrast and brightness, but no other digital enhancements were applied.

### Quantification and statistical analysis

Values were measured from the acquired data with Clampfit 10.7 (Molecular Devices). Statistical analyses were made with Excel 2016 (Microsoft) and R programming software. The time course of the measured values was presented as mean ± standard deviation in figures. Then distributions of measured data of compared groups were presented with Tukey method box and whisker graphs. Two-way ANOVA with repeated measures was used to compare the spike-current relationship between Z+ and Z- PCs, before and after tetanization. Unpaired Student's t-test was used to compare AHP amplitudes, spike count change, and EPSP amplitude change between Z+ and Z- PCs. Paired Student's t-test was used to compare AHP amplitudes and series resistance before versus after tetanization. Mann-Whitney U test was used to compare spike count increase between Z+ and Z- PCs. Pearson's r test was used to test the correlation between maximum spike count and AHP intensity.

## Data Availability

All data supporting the findings of this study are provided in the main text or supplemental information. The Excel file that contains all original data of experiments and statistics details has been uploaded in the Mendeley Data site (Mendeley Data: https://doi.org/10.17632/bm2gk232mt.1). This study did not generate a novel program code.

## References

[bib1] Abraham W.C. (2008). Metaplasticity: tuning synapses and networks for plasticity. Nat. Rev. Neurosci..

[bib2] Aoki S., Coulon P., Ruigrok T.J.H. (2019). Multizonal cerebellar influence over sensorimotor areas of the rat cerebral cortex. Cereb. Cortex..

[bib3] Barmack N.H., Qian Z.-Y., Kim H., Yoshimura J. (2001). Activity-dependent distribution of protein kinase C-δ within rat cerebellar Purkinje cells following unilateral labyrinthectomy. Exp. Brain Res..

[bib4] Belmeguenai A., Hosy E., Bengtsson F., Pedroarena C.M., Piochon C., Teuling E., He Q., Ohtsuki G., De Jeu M.T., Elgersma Y. (2010). Intrinsic plasticity complements long-term potentiation in parallel fiber input gain control in cerebellar Purkinje cells. J. Neurosci..

[bib5] Bouvier G., Higgins D., Spolidoro M., Carrel D., Mathieu B., Léna C., Dieudonné S., Barbour B., Brunel N., Casado M. (2016). Burst-dependent bidirectional plasticity in the cerebellum is driven by presynaptic NMDA receptors. Cell. Rep..

[bib6] Brochu G., Maler L., Hawkes R. (1990). Zebrin II: a polypeptide antigen expressed selectively by Purkinje cells reveals compartments in rat and fish cerebellum. J. Comp. Neurol..

[bib7] Canto C.B., Broersen R., De Zeeuw C.I. (2018). Intrinsic excitement in cerebellar nuclei neurons during learning. Proc. Natl. Acad. Sci. U S A.

[bib8] Cerminara N.L., Lang E.J., Sillitoe R.V., Apps R. (2015). Redefining the cerebellar cortex as an assembly of non-uniform Purkinje cell microcircuits. Nat. Rev. Neurosci..

[bib9] Disterhoft J.F., Oh M.M. (2006). Learning, aging and intrinsic neuronal plasticity. Trends Neurosci..

[bib11] Fujita H., Aoki H., Ajioka I., Yamazaki M., Abe M., Oh-Nishi A., Sakimura K., Sugihara I. (2014). Detailed expression pattern of aldolase C (Aldoc) in the cerebellum, retina and other areas of the CNS studied in Aldoc-Venus knock-in mice. PLoS One.

[bib13] Grasselli G., He Q., Wan V., Adelman J.P., Ohtsuki G., Hansel C. (2016). Activity-dependent plasticity of spike pauses in cerebellar Purkinje cells. Cell Rep..

[bib14] Grasselli G., Boele H.J., Titley H.K., Bradford N., van Beers L., Jay L., Beekhof G.C., Busch S.E., De Zeeuw C.I., Schonewille M., Hansel C. (2020). SK2 channels in cerebellar Purkinje cells contribute to excitability modulation in motor learning-specific memory traces. PLoS Biol..

[bib15] Hawkes R. (2014). Purkinje cell stripes and long-term depression at the parallel fiber-Purkinje cell synapse. Front. Syst. Neurosci..

[bib16] Ito M. (2012).

[bib17] Kaneko R., Takatsuru Y., Morita A., Amano I., Haijima A., Imayoshi I., Tamamaki N., Koibuchi N., Watanabe M., Yanagawa Y. (2018). Inhibitory neuron-specific Cre-dependent red fluorescent labeling using VGAT BAC-based transgenic mouse lines with identified transgene integration sites. J. Comp. Neurol..

[bib18] Kostadinov D., Beau M., Pozo M.B., Hausser M. (2019). Predictive and reactive reward signals conveyed by climbing fiber inputs to cerebellar Purkinje cells. Nat. Neurosci..

[bib19] Kourrich S., Calu D.J., Bonci A. (2015). Intrinsic plasticity: an emerging player in addiction. Nat. Rev. Neurosci..

[bib20] Malhotra S., Banumurthy G., Pennock R., Vaden J.H., Sugihara I., Overstreet-Wadiche L., Wadiche J.I. (2021). Climbing fiber-mediated spillover transmission to interneurons is regulated by EAAT4. J. Neurosci..

[bib21] McEchron M.D., Disterhoft J.F. (1997). Sequence of single neuron changes in CA1 hippocampus of rabbits during acquisition of trace eyeblink conditioned responses. J. Neurophysiol..

[bib22] McKay B.M., Matthews E.A., Oliveira F.A., Disterhoft J.F. (2009). Intrinsic neuronal excitability is reversibly altered by a single experience in fear conditioning. J. Neurophysiol..

[bib23] Nguyen-Minh V.T., Tran-Anh K., Luo Y., Sugihara I. (2019). Electrophysiological excitability and parallel fiber synaptic properties of Zebrin-Positive and -Negative Purkinje cells in Lobule VIII of the mouse cerebellar slice. Front. Cell Neurosci..

[bib24] Paukert M., Huang Y.H., Tanaka K., Rothstein J.D., Bergles D.E. (2010). Zones of enhanced glutamate release from climbing fibers in the mammalian cerebellum. J. Neurosci..

[bib25] Person A.L., Raman I.M. (2011). Purkinje neuron synchrony elicits time-locked spiking in the cerebellar nuclei. Nature.

[bib26] Pijpers A., Apps R., Pardoe J., Voogd J., Ruigrok T.J. (2006). Precise spatial relationships between mossy fibers and climbing fibers in rat cerebellar cortical zones. J. Neurosci..

[bib27] Piochon C., Titley H.K., Simmons D.H., Grasselli G., Elgersma Y., Hansel C. (2016). Calcium threshold shift enables frequency-independent control of plasticity by an instructive signal. Proc. Natl. Acad. Sci. U S A.

[bib28] Rosse C., Linch M., Kermorgant S., Cameron A.J., Boeckeler K., Parker P.J. (2010). PKC and the control of localized signal dynamics. Nat. Rev. Mol. Cell Biol..

[bib29] Sarpong G.A., Vibulyaseck S., Luo Y., Biswas M.S., Fujita H., Hirano S., Sugihara I. (2018). Cerebellar modules in the olivo-cortico-nuclear loop demarcated by pcdh10 expression in the adult mouse. J. Comp. Neurol..

[bib30] Schreurs B.G., Gusev P.A., Tomsic D., Alkon D.L., Shi T. (1998). Intracellular correlates of acquisition and long-term memory of classical conditioning in Purkinje cell dendrites in slices of rabbit cerebellar lobule HVI. J. Neurosci..

[bib31] Shim H.G., Jang D.C., Lee J., Chung G., Lee S., Kim Y.G., Jeon D.E., Kim S.J. (2018). Long-term depression of intrinsic excitability accompanied by synaptic depression in cerebellar. Purkinje cells. J. Neurosci..

[bib32] Sugihara I., Shinoda Y. (2004). Molecular, topographic, and functional organization of the cerebellar cortex: a study with combined aldolase C and olivocerebellar labeling. J. Neurosci..

[bib33] Sugihara I., Marshall S.P., Lang E.J. (2007). Relationship of complex spike synchrony bands and climbing fiber projection determined by reference to aldolase C compartments in crus IIa of the rat cerebellar cortex. J. Comp. Neurol..

[bib34] Suzuki L., Coulon P., Sabel-Goedknegt E.H., Ruigrok T.J. (2012). Organiza-tion of cerebral projections to identified cerebellar zones inthe posterior cerebellum of the rat. J. Neurosci..

[bib35] Titley H.K., Brunel N., Hanse l C. (2017). Toward a neurocentric view of learning. Neuron.

[bib36] Titley H.K., Watkins G.V., Lin C., Weiss C., McCarthy M., Disterhoft J.F., Hansel C. (2020). Intrinsic excitability increase in cerebellar Purkinje cells after delay eye-blink conditioning in mice. J. Neurosci..

[bib37] Tran-Anh K., Zhang J., Nguyen-Minh V.T., Fujita H., Hirata T., Sugihara I. (2020). Common Origin of the cerebellar dual somatotopic areas revealed by tracking embryonic Purkinje cell clusters with birthdate tagging. eNeuro.

[bib38] Tsutsumi S., Yamazaki M., Miyazaki T., Watanabe M., Sakimura K., Kano M., Kitamura K. (2015). Structure-function relationships between aldolase C/zebrin II expression and complex spike synchrony in the cerebellum. J. Neurosci..

[bib39] Tsutsumi S., Hidaka N., Isomura Y., Matsuzaki M., Sakimura K., Kano M., Kitamura K. (2019). Modular organization of cerebellar climbing fiber inputs during goal-directed behavior. eLife.

[bib40] Voogd J., Ruigrok T.J.H. (2004). The organization of the corticonuclear and olivocerebellar climbing fiber projections to the rat cerebellar vermis: the congruence of projection zones and the zebrin pattern. J. Neurocytol..

[bib41] Wadiche J.I., Jahr C.E. (2005). Patterned expression of Purkinje cell glutamate transporters controls synaptic plasticity. Nat. Neurosci..

[bib42] Wang D.J., Su L.D., Wang Y.N., Yang D., Sun C.L., Zhou L., Wang X.X., Shen Y. (2014). Long-term potentiation at cerebellar parallel fiber-Purkinje cell synapses requires presynaptic and postsynaptic signaling cascades. J. Neurosci..

[bib43] Wang D., Smith-Bell C.A., Burhans L.B., O'Dell D.E., Bell R.W., Schreurs B.G. (2018). Changes in membrane properties of rat deep cerebellar nuclear projection neurons during acquisition of eyeblink conditioning. Proc. Natl. Acad. Sci. U S A.

[bib44] Widmer H.A., Rowe I.C.M., Shipston M.J. (2003). Conditional protein phosphorylation regulates BK channel activity in rat cerebellar Purkinje neurons. J. Physiol..

[bib45] Wu B., Blot F.G., Wong A.B., Osório C., Adolfs Y., Pasterkamp R.J., Hartmann J., Becker E.B., Boele H.J., De Zeeuw C.I., Schonewille M. (2019). TRPC3 is a major contributor to functional heterogeneity of cerebellar Purkinje cells. eLife.

[bib46] Xiao J., Cerminara N.L., Kotsurovskyy Y., Aoki H., Burroughs A., Wise A.K., Luo Y., Marshall S.P., Sugihara I., Apps R., Lang E.J. (2014). Systematic regional variations in Purkinje cell spiking patterns. PLoS One.

[bib47] De Zeeuw C.I., Ten Brinke M.M. (2015). Motor learning and the cerebellum. Cold Spring Harb. Perspect. Biol..

[bib48] Zhou H., Lin Z., Voges K., Ju C., Gao Z., Bosman L.W., Ruigrok T.J., Hoebeek F.E., De Zeeuw C.I., Schonewille M. (2014). Cerebellar modules operate at different frequencies. eLife.

