# Supplementary material for: Heterogeneity of intrinsic plasticity in cerebellar Purkinje cells linked with cortical molecular zones
Source: iScience. 2021 Dec 28;25(1):103705. doi: 10.1016/j.isci.2021.103705 (PMC8760437; doi:10.1016/j.isci.2021.103705)
Supplement: Document S1. Figures S1–S5 [file mmc1.pdf]

iScience, Volume *25*

Supplemental information

Heterogeneity of intrinsic plasticity in cerebellar Purkinje cells linked with cortical molecular zones

Nguyen-Minh Viet, Tianzhuo Wang, Khoa Tran-Anh, and Izumi Sugihara

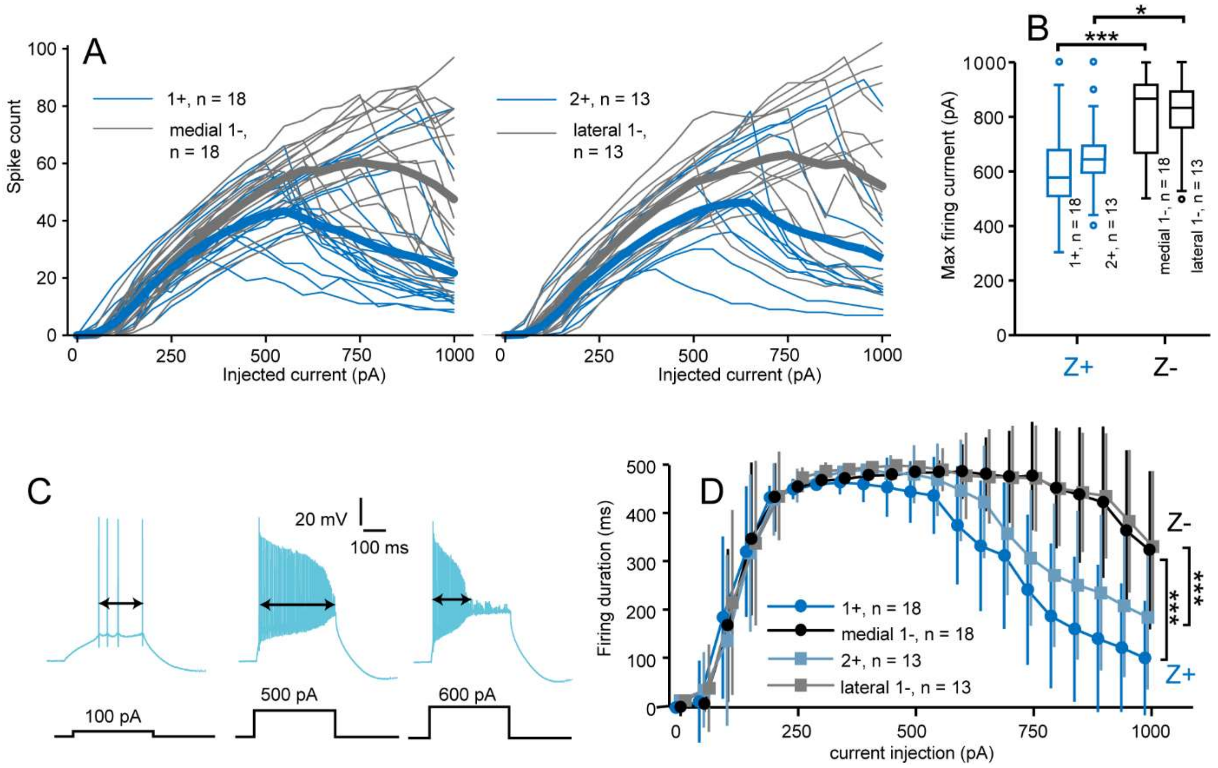

Figure S1. Further comparison of the intrinsic excitability between Z+ and Z- PCs in neighboring zones. Related to Figure 1.

(**A**) Spite-current relationship of individual PCs up to 1000 pA current injection. Thick lines indicate the average of Z+ (blue) and Z- (black) PCs. (**B**) Intensity of the injected current that produced the maximum number of spikes in a PC (maximum firing current). (**C**) Samples spike responses to square current injection (100, 500, and 600 pA for 500 ms) to illustrate the firing duration (time between first and last spikes). (**D**) Firing duration plotted against the intensity of the injected current. Data shown in this figure were obtained from the same recordings as used in Figure 1. Data are presented as mean ± standard deviation in (**D**). The number of Z+ and Z- PCs, 18 in 1+, 18 in medial 1-, 13 in lateral 1-, and 13 in 2+. The significant difference was tested with two-way ANOVA with repeated measures in (**D**, 1+/medial 1-, $F(1,714)=129.2$, $p=0.0000$, n=18, 18; 2+/lateral 1-, $F(1,504)=58.10$, $p=0.0000$, n=13, 13) and unpaired Student t-test in (**B**, 1+/medial 1-, $t(34)=-3.732$, $p=0.00069$, n=18, 18; 2+/lateral 1-, $t(24)=-2.3933$, $p=0.0249$, n=13, 13). ***$p<0.001$, *$p < 0.05$. All recorded PCs were located in zones 1+, 1- and 2+ in lobule IV-V.

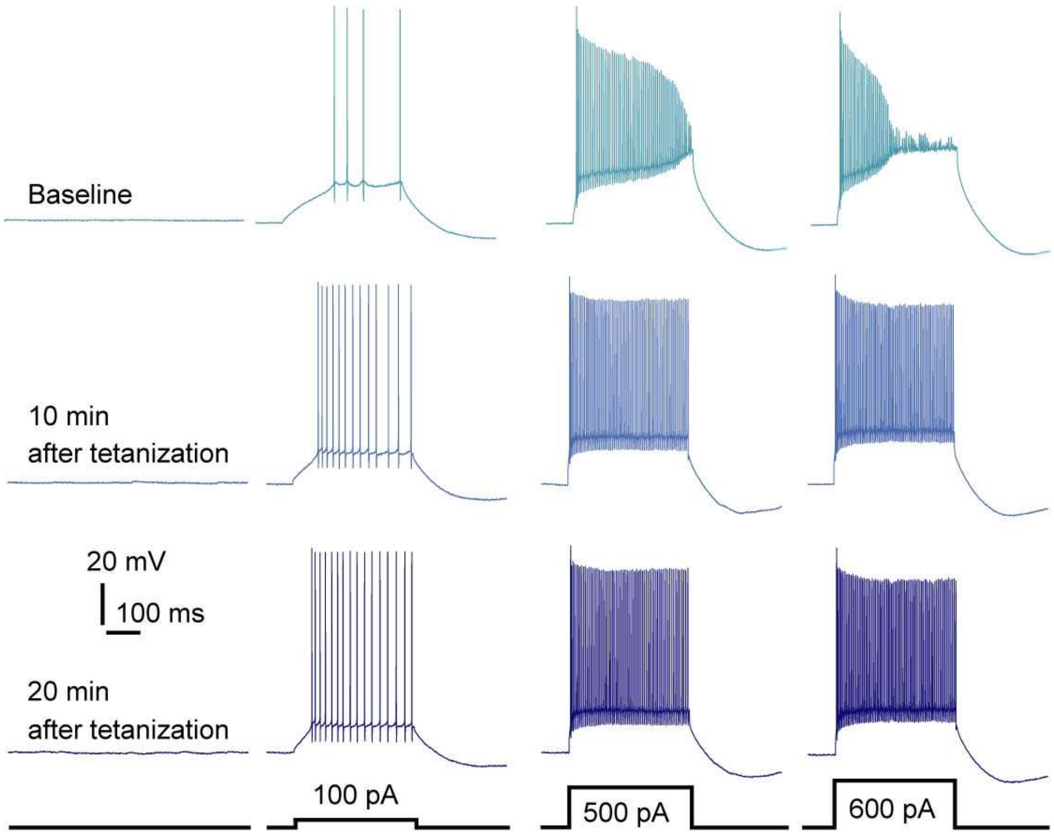

Figure S2. Original spike recordings under the current clamp showing intrinsic plasticity with an enhancement of spike count in a Z- PC. Related to Figure 3.

Before applying the tetanizing stimulation of the LTP-IE protocol (baseline), a decrease of spike count was observed at 600 pA current injection due to intrinsic depolarization block. After the tetanization, the PC became more capable of spiking continuously at 500 pA and 600 pA current injections with no intrinsic depolarization block.

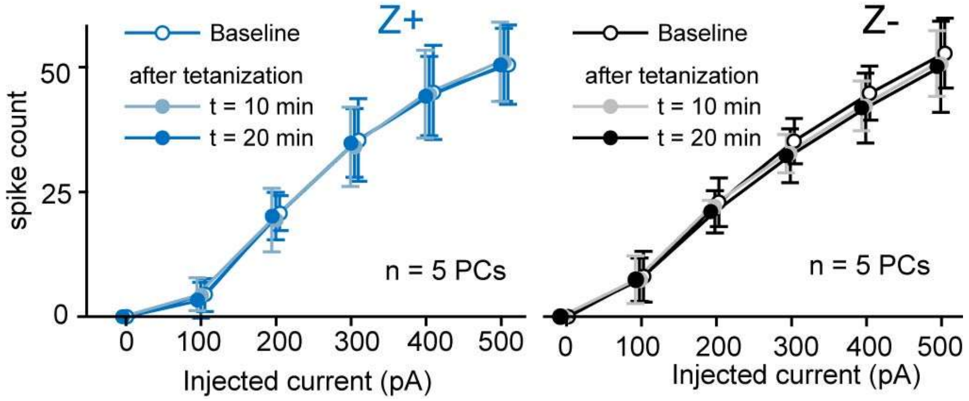

Figure S3. Stable current-spike relationship in a Z+ and Z- PCs control condition. Related to Figure 3.

As the control for the LTP-IE protocol (cf. Fig. 3), we measured the spike-current relationship in the exact timing (initial measurement and two measurements 10 and 20 minutes after the first measurement) without giving the tetanization. Then the spike-current relationship was relatively stable for 20 min. Data are represented as mean ± standard deviation. No significant difference was found with two-way ANOVA with repeated measures (before and 20 min after; Z+, $F(1,48)=0.091$, $p=0.764$, n=5, 5; Z-, $F(1,48)=1.492$, $p=0.228$, n=5, 5). All recorded PCs were located in zones 1+, 1- and 2+ in lobule IV-V.

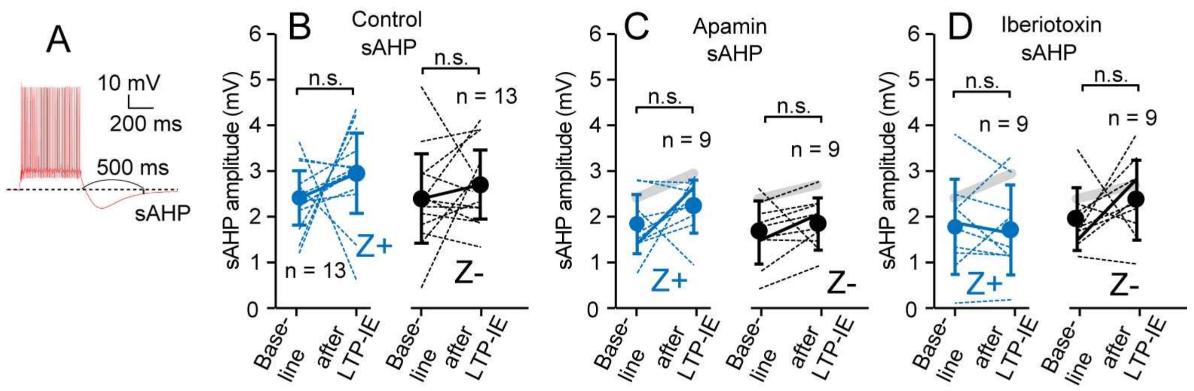

Figure S4. Slow after hypopolarization (sAHP) did not show a significant change in LTP-IE in either Z+ or Z- PCs in control condition or under apamin or iberiotoxin administration. Related to Figures 3 and 4.

(**A**) Illustration of measurement of sAHP amplitude. (**B-D**) Change in the sAHP amplitude in LTP-IE. Amplitudes of the sAHP in individual PCs (dashed lines) and the average (solid line) were compared before and 20 minutes after the tetanization in Z+ (left graph in each panel) and Z- (right graph in each panel) PCs in the control condition (**B**), under apamin (**C**) and iberiotoxin (**D**). Pale gray lines in (**C**) and (**D**) indicate the average change under the control condition (**B**). Summary data are represented as mean ± standard deviation. in (**B, C, D**). The Student's paired t-test was used (**B**, Z+, t(12)=-1.175, p=0.263n=13; Z-, t(12)=-0.882, p=0.395, n=13; **C**, Z+, t(8)=-1.301, p=0.229, n=9; Z-, t(8)=-0.906, p=0.391, n=9; **D**, Z+, t(8)=0.282, p=0.785, n=9; Z-, t(8)=-1.083, p=0.310, n=9). n.s., p > 0.05. All recorded PCs were located in zones 1+, 1- and 2+ in lobule IV-V.

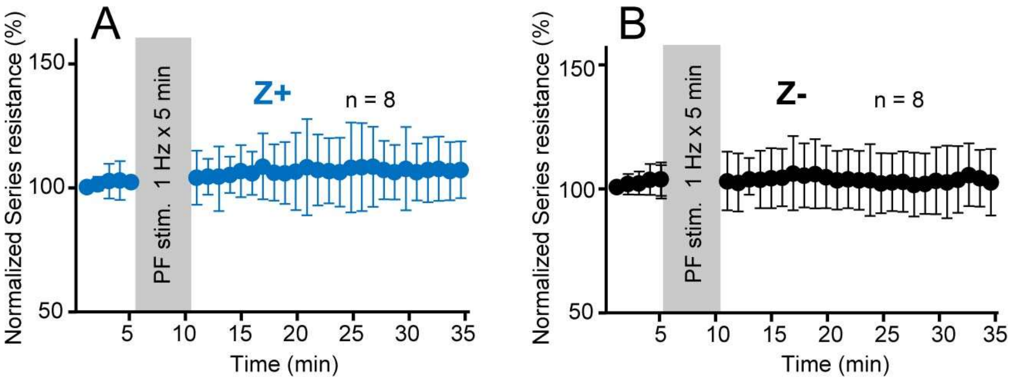

Figure S5. Series resistance stability in synaptic LTP experiments. Related to Figure 5.

Time graph of the normalized series resistance before and after the 1-Hz PF stimulation to produce the LTP of the PF-PC synapse in Z+ and Z- PCs (A and B, respectively) in zebrin zones 1+, 1- and 2+ in lobule IV-V. Recorded in the same experiments as shown in Figure 5. Relatively stable series resistance was confirmed. Data are represented as mean ± standard deviation. Comparison of the average of the series resistances before and 21-25 minutes after the 1-Hz PF stimulation produced no significant difference with paired Student's t-test (A, $t(7)=1.755$, $p=0.123$, $n=8$; B, $t(7)=0.395$, $p=0.704$, $n=8$).
